# Statistical analysis and molecular dynamics simulations of ambivalent *α *-helices

**DOI:** 10.1186/1471-2105-11-519

**Published:** 2010-10-18

**Authors:** Nicholus Bhattacharjee, Parbati Biswas

**Affiliations:** 1 Department of Chemistry, University of Delhi, Delhi - 110007, India

## Abstract

**Background:**

Analysis of known protein structures reveals that identical sequence fragments in proteins can adopt different secondary structure conformations. The extent of this conformational diversity is influenced by various factors like the intrinsic sequence propensity, sequence context and other environmental factors such as pH, site directed mutations or alteration of the binding ligands. Understanding the mechanism by which the environment affects the structural ambivalence of these peptides has potential implications for protein design and reliable local structure prediction algorithms. Identification of the structurally ambivalent sequence fragments and determining the rules which dictate their conformational preferences play an important role in understanding the conformational changes observed in misfolding diseases. However, a systematic classification of their intrinsic sequence patterns or a statistical analysis of their properties and sequence context in relation to the origin of their structural diversity have largely remained unexplored.

**Results:**

In this work, the conformational variability of *α*-helices is studied by mapping sequences from the non-redundant database to identical sequences across all classes of the SCOP (Structural Classification of Proteins) database. Some helices retain their conformations when mapped in the SCOP database while others exhibit a complete/partial switch to non-helical conformations. The results clearly depict the differences in the propensities of amino acids for the variable and conserved helices. Sequences flanking these ambivalent sequence fragments have anisotropic propensities at the N- and C-termini. This structural variability is depicted by molecular dynamics simulations in explicit solvent, which show that the short conserved helices retain their conformations while their longer counterparts fray into two or more shorter helices. Variable helices in the non-redundant database exhibit a trend of retaining helical conformations while their corresponding non-helical conformations in SCOP database show large deviations from their respective initial structures by adopting partial or full helical conformations. Partially ambivalent helices are also found to retain their respective conformations.

**Conclusions:**

All sequence fragments which show structural diversity in different proteins of the non-redundant database are investigated. The final conformation of these ambivalent sequences are dictated by a fine tuning of their intrinsic sequence propensity and the anisotropic amino acid propensity of the flanking sequences. This analysis may unravel the connection between diverse secondary structures, which conserve the overall structural fold of the protein thus determining its function.

## Background

Conformational variability in proteins arises from a subtle interplay of a combination of environmental factors and intrinsic propensity of amino acids in different sequence contexts. This diversity often provides a route for monitoring protein activation and permits functional promiscuity. The magnitude of conformational diversity noted in proteins ranges from the side-chain fluctuations to a partial/complete change in secondary structures and even rearrangements of the tertiary structure. Various terms are used to describe this phenomenon [1-6] and can be confirmed with the availability of data from various related disciplines like protein folding, NMR and fast kinetics. It is a well established that the local sequence-to-structure mapping is not one to one over the entire sequence space [7-9] though there are numerous examples of highly structurally conserved local sequence patterns. Certain type of sequences can adopt either an *α*-helical or a *β*-sheet conformation and a limited number of substitutions can convert an *α*-helical protein to a predominantly *β*-sheet protein [10,11]. Other studies have also demonstrated that several different contexts such as change in pH [12,13], alteration of the binding ligand [14] or site-directed mutations [15,16] induce the structural transition between an *α*-helix and a *β*-strand or random coil. It has been confirmed that this conformational switch from *α*-helix to *β*-sheet/*β*-hairpin structure plays a significant role in the misfolding diseases as in amyloid fibril formation [17,18]. A detailed analysis of the relative magnitudes of the context-dependent factors on the conformational preferences of these ambivalent sequence fragments is important for reliable local structure prediction.

Both experiments and statistical analysis [19-28] confirm that different amino acids have different propensities for *α*-helix or *β*-strand formation. Quantifying these propensity scales provides local sequence information for predicting secondary structures. However, both experimental and theoretical study [10,29-31] have shown that the peptides having identical sequences may adopt different secondary structures in different proteins. Determining the rules which govern the structural ambivalence of these sequences and analyzing the contribution of intrinsic propensity, sequence context and environmental factors to the conformational preference of such sequences may have important implications in the pathogenesis of amyloid diseases including Alzheimer disease and designing *de novo *proteins. Ambivalent sequences are also suggested to be one of the reasons behind upper limit of prediction accuracy for secondary structure prediction [32].

The structurally ambivalent sequences were first reported by Kabsch and Sander [8] who predicted protein structures based on sequence homology. They investigated the structural significance and adaptability of short sequence homologies by searching 62 proteins of known three-dimensional structures. These sequentially identical proteins adopt different secondary structures, each sequence occurs once as an *α*-helix and once as a *β*-strand. Subsequent studies [9,33-36] confirmed this observation by scanning a larger database with lower percentage of sequence identity. However, a systematic identification and classification of the sequence patterns, conformational preferences of these structurally ambivalent segments and their corresponding flanking residues largely remain unexplored.

This work aims to assess the degree of conformational variability of these ambivalent sequence segments quantitatively in known protein structures and examines the factors that affect their respective preferences for a particular type of backbone conformation. In this work, we analyze the *α*-helices (since *α*-helices are considered to show higher conformational diversity than *β*-sheets [35]) from non-redundant database and map them to proteins belonging to all classes of SCOP database to find identical sequences. Earlier studies have shown that ambivalent sequences arise from different structural classes [33,36]. In this study, we have mapped helical sequences generated from a non-redundant data base into different SCOP classes to find helices which are conserved in certain proteins but change into non-helical structures in others. Unlike previous studies we have considered a relatively wide range of sequence lengths, both short and long to portray the pattern of variation of the different physico-chemical properties from the conserved helices to the variable helices, i.e., those which have different conformations in different proteins. We also identify the helical sequences which partially switch their conformations. Although partially ambivalent sequences were reported earlier, no detailed analysis of their physico-chemical properties are done. To our knowledge this is the first detailed analysis, which reflects the trend of variation of the different physico-chemical properties ranging from the conserved to variable helices through partially variable helices. The residues flanking the helical and their corresponding non-helical sequences are also analysed to record anisotropic amino acid distributions in the N- and C-termini. Most of the conserved and some of the variable helices are found to adopt the same fold in both non-redundant and SCOP database. Detailed molecular dynamics simulation results show that the variable helices retain their helical conformations after simulation. The corresponding non-helical conformations show large deviations from their initial structure by adopting helical or partially helical conformations. The short conserved helices are found to retain their conformations while longer conserved helices fray into two or more number of shorter helices. The selection of a large database makes the results free of database biases and inconsistent parameters.

## Results and Discussion

### Population of helices with different degree of conformational variation

**Figure 1:**
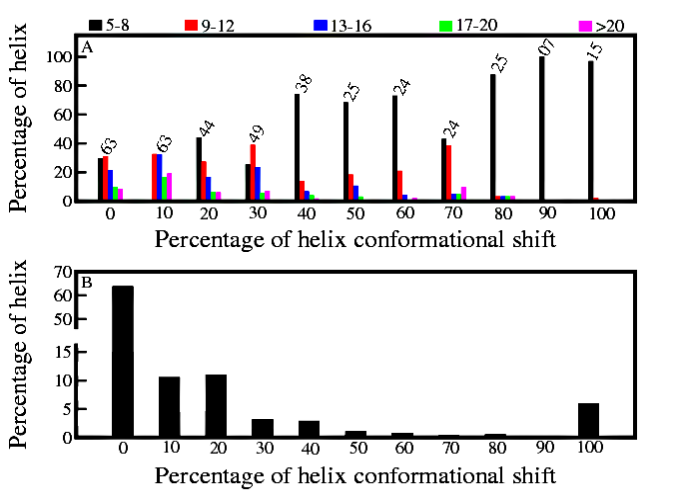
**Length Distribution vs. Conformational Shift**. (A) Length distribution of the helices versus the percentage conformational shift. The numbers above the bars show the length of the longest helix in that group. The color code depicts the length range. (B) Fraction of helices (out of 6338) present in different percentage conformational shift bins.

The May 2008 release of PDB select [37] consists of 11592 helices. Of these, 6338 helices are mapped on to the SCOP database (release 1.73) [38] with different degree of conformational variability. Length distribution of these helices plotted against the percentage conformational transition in the SCOP database in Figure 1A reveals that the shorter peptide sequences (≤ 15 residues long) switch readily from helical to non-helical conformations. Since the longer helices consist of more hydrogen bonds, the transition to non-helical conformations is energetically expensive, leading to the conservation of these helices in the chosen database. Figure 1B shows the number of helices in % (out of 6338) in different conformational shift bins. The Figure shows that a large number of helices (more than 60%) are conserved helices and record no conformational shift (0% shift). The number of helices decreases as extent of conformational shift increases with very few helices beyond 30% conformational shift (approximately 3% of the total number of helices). However, number of variable helices with 100% conformational shift are comparable to the number of helices present in the 10% and 20% conformational shift bins (~10%). Detailed analysis shows that for conserved helices (helices with 0% conformational shift) the sequences are in same SCOP fold for both non-redundant and corresponding SCOP database in 92% cases and in same SCOP domain in 90% cases. The corresponding values for variable helices (helices with 100% conformational shift) are 7% and 6% respectively.

### Conserved and variable helices have different preference of amino acids

**Figure 2:**
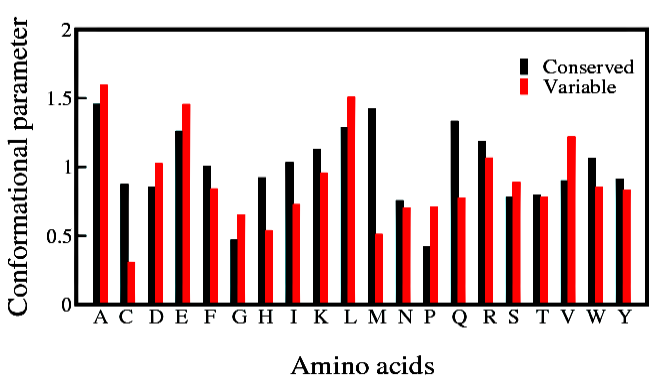
**Conformational Parameters of Amino Acids in Conserved and Variable helices**. Relative frequency of occurrences of the amino acids in the conserved and variable helices depicted as conformational parameters.

Conserved helices always retain their structures when they are mapped from the non-redundant database to different SCOP classes while the variable helices undergo a complete transition to non-helical conformations in the SCOP database. The relative frequency of occurrence of the *i^th ^*amino acid in variable/conserved helices *j *is depicted as normalized conformational parameter (*CP_ij_*) in Figure 2. This normalization is with respect to the frequency of occurrence of the corresponding amino acid in the non-redundant database.

(1) $$CP_{ij}=\frac{f_{ij}}{f_{i}}=\frac{n_{ij}/\sum_{i=1}^{20}n_{ij}}{N_{i}/\sum_{i=1}^{20}N_{i}}$$

where *n_ij _*and *f_ij _*are the number and fraction of finding *i^th ^*amino acid in given secondary structure *j *while *N_i _*and *f_i _*are the number and fraction of finding *i^th ^*amino acid in the non-redundant database. According to Chou-Fasman scale [39], M, Q, W are helix forming residues which show a distinct propensity for conserved helices as compared to the variable helices, while V reveals a completely opposite trend. For all other amino acids conformational parameters for both conserved and variable helices are either higher or lower than 1, reflecting respective preference or aversion. Although bulky side chain of W was hypothesised to be the cause behind its low frequency in ambivalent helical sequences [36], the reason behind their low occurrence in these type of sequences is yet to be fully understood. It might be possible that high occurrences of M, Q, W in conserved helices impart extra stability, which is not present in case of variable helices, leading to a change in their conformation.

In accordance to the earlier observations, A, I, L, V [34-36] prefer to occur in variable helical sequences. These residues have unspecific hydrophobic interactions, which permit a greater number of possible orientations in a hydrophobic environment and they are structurally ambivalent. Variable helices have high frequency of G and P in comparison to the conserved helices. These residues are considered to be strong helix disruptors and hence their higher frequency of occurrence in variable helices is clearly understood. Cysteines tend to form disulfide bonds imparting higher stability to the sequence fragment. Cysteines have a very low frequency of occurrence in variable helices rendering the flexibility needed for the transition to non-helical structures. This observation is consistent with the earlier studies [34-36]. Frequencies of occurrence of other amino acids are also found to be similar with the previous results [34-36]. However, Aspartic and Glutamic acids show notable deviations. Both these amino acids are found to have higher frequency of occurrence in variable helices compared to conserved helices which is in conflict with the earlier observations [34-36]. In both conserved and variable helices, the propensity of Glutamic acid is higher (*CP_ij _*≥ 1) than that of Aspartic acid (*CP_ij _*≤ 1), which dictates their intrinsic preference for helices [39].

### Conformational parameter of amino acids varies with respect to the percentage ambivalency of the helical sequences

**Figure 3:**
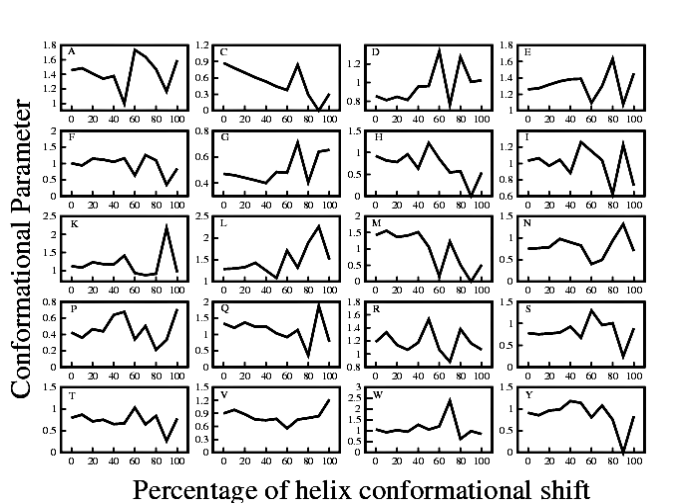
**Conformational Parameter vs. Percentage Ambivalency**. Conformational parameters of the amino acids in the ambivalent sequences plotted as a function of their respective percentage ambivalency. Upper left part of each graph gives the one letter abbreviation of amino acid for which the graph is drawn.

Though partially ambivalent sequences are reported earlier [34,35,40], but surprisingly there is no detailed analysis of their sequence properties. Here we have performed a systematic analysis of the amino acid distribution in different sequences with different degrees of ambivalency depicted as the percentage conformational shift from helix to non-helical structures. Figure 3 depicts the conformational parameters of all amino acids in the ambivalent sequences plotted as a function of their respective percentage ambivalency. It is interesting to note that up to approximately 50% conformational shift, the values of the conformational parameters of all amino acids remain almost similar. C, G, M, P exhibit maximum deviation in the conformational parameter values above 50% structural ambivalency. G and P are the helix breaking amino acids which exhibit a considerable increase in the values of conformational parameters when 50% residues of the helix change to a non-helical conformation. Cysteines are found to decrease rapidly when 50% conformational shift occurs from a helical structure. A very similar trend is observed for Methionine.

### Flanking sequences have different distributions of the amino acids near the two termini of variable helices

**Figure 4:**
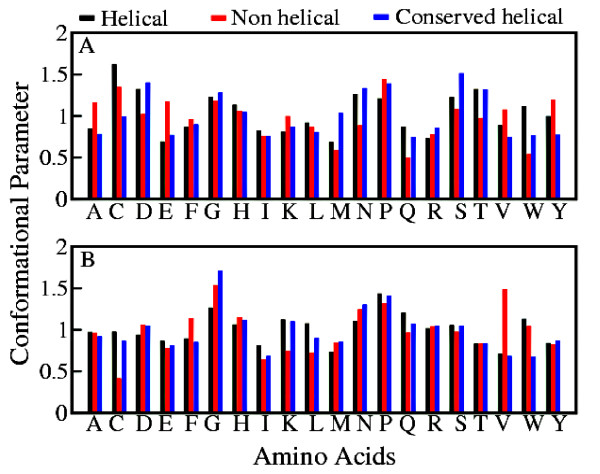
**Conformational Parameter in Flanking Sequences**. Conformational parameter of amino acids in flanking sequences towards (A) N-terminus and (B) C-terminus of helical and non- helical conformations of variable helices as well as of conserved helices.

Flanking sequences follow different amino acid distribution patterns for the helical and the corresponding non-helical conformations in variable helices. The flanking residues play an important role in determining the conformation of ambivalent sequences [34-36]. This has been also demonstrated experimentally [10]. Here we have considered up to four residues flanking each termini of the variable helices to plot the distribution of the flanking amino acids for N- and C-termini in Figure 4. To our knowledge this is the first report where the flanking amino acids are differentiated as N-terminus flanking and C-terminus flanking residues. Amino acids follow distinctly different distribution patterns in both flanks of the variable helices. Since helices are initiated and terminated by different amino acids, a difference in the amino acid distribution in two flanks of the ambivalent sequences is not surprising. A common example is Alanine, whose frequency of occurrence is higher in residues flanking N-terminus of non-helical conformations than in helical conformations whereas in the C-terminus flanking sequences it has almost similar frequency of occurrence for both helical as well as non-helical conformations. A completely opposite trend can be observed in case of Glycine. This non-equivalence termini dependent difference in the distribution patterns of the flanking residues of helices and non-helical structures may be observed for other amino acids too. In accordance to earlier studies [34-36] Glycine and Proline are found to have high preferences (*CP_ij _*> 1) for sequences flanking ambivalent helices establishing their role as helix breakers. The frequency of most of the other amino acids in the flanking sequences are different to that found in similar studies on chameleon sequences [34-36]. However, it should be noted that the method of determining ambivalent sequences is quite different in this analysis as compared to the earlier ones. Previous studies [34-36] highlighted similar sub-sequences which are found to adopt both helix and strand conformations in different proteins of the non-redundant database, while in this work the helical sequences of varying lengths found in the non-redundant database of proteins are mapped into various SCOP classes to analyze the pattern of partial/complete conservation/variation across the same folds. This study may have a significant implication on the structural diversity linked with the conservation of the function or fold. We have also plotted the conformational parameters of the amino acids in the flanking sequences of the conserved helices in Figures 4 for comparison. Except Cysteine and Methionine at N-terminus and Tryptophan at C-terminus all other amino acids have similar values of conformational parameters for the flanking sequences of both the variable and conserved helices.

### Flanking sequences possess different environment

**Figure 5:**
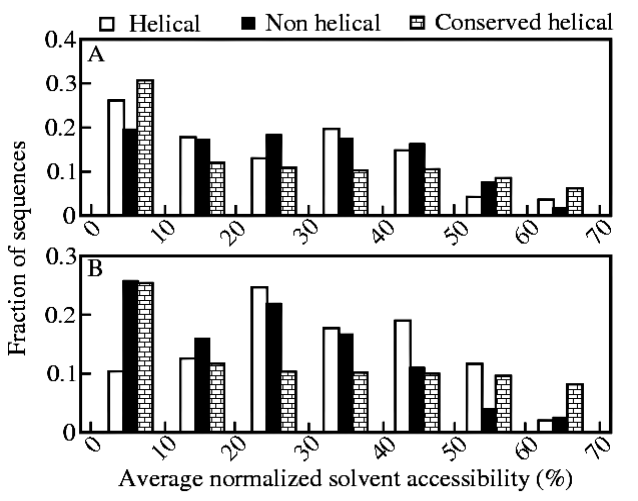
**Normalized Solvent Accessibility**. Fraction of (A) N-terminus and (B) C-terminus flanking sequences of helical and non-helical conformations of variable helices as well as of conserved helices with different average normalized solvent accessibility.

Variable helices in both helical and non-helical conformations are found to possess similar solvent accessibility which is in accordance with the earlier studies [33]. To explore the local environment of the residues, the solvent accessibility of the sequences flanking helices and non-helical conformations is determined. The solvent accessibility for a given residue X is calculated with the DSSP software [41], which is normalised with respect to the maximum solvent accessibility found in Gly-X-Gly. Figure 5 depicts the fraction of these flanking sequences with average normalised solvent accessibility. However, it is rather interesting to note that the flanking residues have different solvent environments for helical and non-helical conformations towards N- and C-termini. Residues flanking N-terminus of helices have lower solvent accessibility than its analogue in non-helical conformations, while a completely opposite trend may be observed for the C-terminus flanking residues. For the sake of comparison, we have also plotted fraction of sequences flanking conserved helices with respect to different average normalised solvent accessibility in Figure 4. Variable and conserved helices exhibit a similar pattern towards N-terminus while they are completely different towards C-terminus.

### Variable helical sequences try to retain their helical conformation

Molecular dynamics simulations are performed for a few representative conserved and variable helices with an explicit water model. For variable helices, simulations are performed for both proteins where the particular sequence is in helical and non-helical conformation respectively. These proteins are chosen randomly from the database for simulation such that we have at least one representative protein chain from each SCOP class. Variable helices are simulated by different protocols viz., simulation of the target chain, simulation of the target chain by constraining all other chains, simulation of the whole protein. Most of the results are provided in the Additional file 1 (for 10 nano second simulations) and Additional file 2 (for 1 nano second simulations). The final conformations of the variable and conserved helices are similar for both 10 and 1 nano second simulations which indicate that the conformations corresponding to these sequences have marginal dependence on simulation time. Here we discuss representative simulations both for a variable helix in helical and non-helical conformations and for a conserved helix.

**Figure 6:**
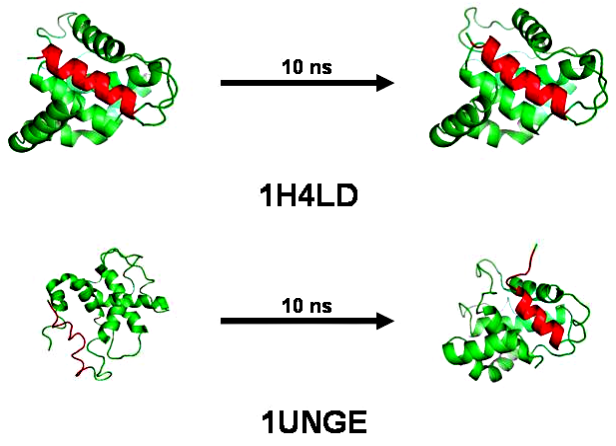
**MD results of Variable Sequences**. Example of an ambivalent sequence (variable helix) both in helical (in 1H4LD) as well as in non-helical (in 1UNGE) conformations (shown in red color). The figures also show the conformations after 10 nano second MD simulation. Figures are generated with pymol software.

**Figure 7:**
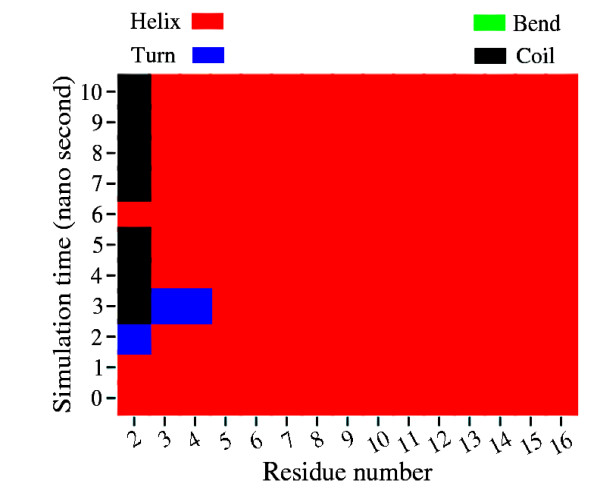
**Time Evolution of Secondary Structures in Variable Helix**. Time evolution of secondary structures of the variable helical sequence in 1H4LD. DSSP annotation is used with H & G as helical, B & E as strand, T as turn, S as bend and others as coil.

**Figure 8:**
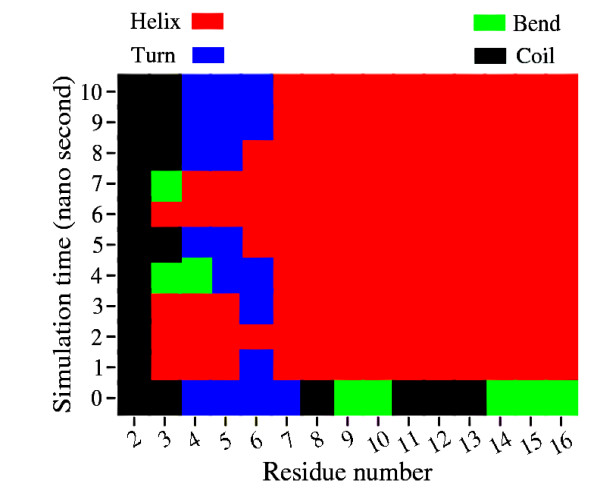
**Time Evolution of Secondary Structures in Variable Non-helix**. Time evolution of secondary structures of the variable helical sequence in 1UNGE. The secondary structure annotations are similar to that of figure 7.

Figure 6 depicts the structures of protein chains 1H4LD and 1UNGE from the non-redundant and SCOP database respectively. Both protein chains belong to SCOP class All Alpha Protein. The sequence fragment 148-162 (TSELLRCLGEFLCRR) in both protein chains are identical. This sequence adopts a helical conformation in 1H4LD while it is found as a random coil in 1UNGE. The helical conformation remains intact after 10 nano seconds of simulation. Figure 7 shows the secondary structure evolution of the helical sequence with respect to time. The final conformation corresponding to this sequence after 1 nano second simulation (refer to Additional file 2) is retained even after 10 nano seconds. This shows that the peptide fragment tries to retain its helical conformation. Similarly, it is found that the variable helices retain their respective conformations after simulation (refer to Additional file 1 and Additional file 2 for more simulation results). More interesting examples are observed for the non-helical segments of ambivalent sequences. For example, the non-helical sequence of 1UNGE shown in Figure 6 adopts a partial helical structure after 10 nano seconds of simulation. Figure 8 depicts how the secondary structures evolve for this sequence fragment with time. This partial helix forms after 1 nano second and this conformation is retained throughout the simulation period. Simulation results (from Additional file 1 and Additional file 2) suggest that the non-helical structures of variable helices assume different conformations after MD simulations with some adopting complete helical conformations, some drifting to partially helical structures and some retaining their initial non-helical conformations.

**Figure 9:**
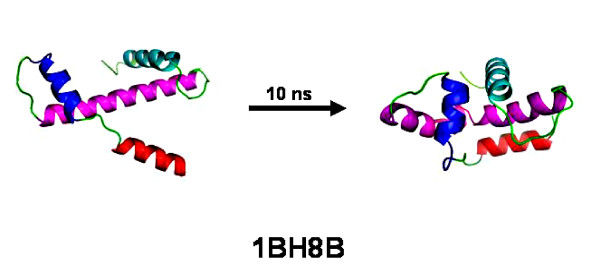
**MD result of Conserved Sequence**. Initial and final structure after 10 nano second MD simulation of protein chain 1BH8B containing four conserved helices (shown in different colors).

**Figure 10:**
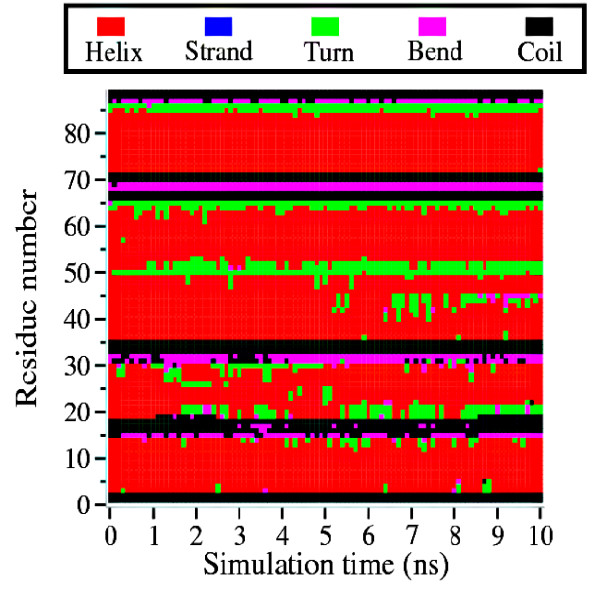
**Time Evolution of Secondary Structures in Protein Having Conserved Helices**. Time evolution of secondary structures for all residues of 1BH8B. Results for four conserved helices individually are shown in Figure S2 (Additional file 1).

In contrast the conformations of conserved helices are found to be length dependent. Figure 9 shows the structure of the protein chain 1BH8B before and after simulation. This protein chain consists of four conserved helices located between residues numbered 3-14 (EEQLNRYEMYRR), 19-30 (KAAIKRLIQSIT), 36-63 (QNVVIAMSGISKVFVGEVVEEALDVCEK) and 72-84 (PKHMREAVRRLKS). Figure 10 shows the time evolution of the helices for all residues of 1BH8B. Secondary structure evolution with respect to time for the four helices separately can be found in Figure S2 (Additional file 1). The results show that the shorter conserved helices are rigid in nature and tend to retain the helical conformation during the simulation period while the longer conserved helices partially break into smaller helices. From Additional file 2 (containing 1 nano second simulation results) it is found that these shorter helices have similar conformation after 1 and 10 nano second simulation. The longer helices show marginal differences in the structures with respect to simulation time. The Zimm-Bragg helix-coil transition theory [42] relates the helix content of a polypeptide to three parameters: s, the intrinsic helix-forming propensity of an amino acid; *σ*, the constant for nucleating the helix; and *n*, the number of peptide units in the polypeptide. Hence, longer helices should be more stable than the shorter ones. However our molecular dynamics studies reveal an exactly opposite trend with the longer conserved helices being more labile compared to the shorter counterparts. This observation may be rationalized by a gain in the conformational entropy which outweighs the favorable energetic interactions in longer helices [43]. Other results provided in Additional file 1 and Additional file 2 also confirm this observation.

Though partially ambivalent sequences are observed previously [34,35,40] no detailed studies on them are reported. Figure S9 and Figure S10 (refer to Additional file 1) depict the behavior of the partially ambivalent helix in proteins 1NQJB and 1NQDB respectively. The sequence LKEKENNDSSDK is a helix at positions 4-15 in the All Beta Protein 1NQJB (by SCOP classification), but looses 75% of its helical structure at positions 7-18 in the partially ambivalent protein chain 1NQDB. After 10 nano seconds of simulation in presence of solvent, the helical conformation in 1NQJB changes to a partial helical structure, while its sequence analogue in 1NQDB, which has a predominantly non-helical conformation remains unchanged. The result shows that the partially ambivalent helix retains its structure and does not drift to a completely non-helical one. The fact that partially ambivalent helices conserve their original structures may be explained by an optimal balance of the energy and conformational entropy associated with the partially helical structures.

## Conclusions

In this study, conserved and variable helices are identified by mapping a given helical sequence from the non-redundant database to identical sequences in the SCOP database. Some helices retain their conformation when mapped in the SCOP database while others exhibit a complete/partial transition to the non-helical conformations. This complete/partial structural variability is depicted by molecular dynamics simulations in explicit solvent which reveal that the helical conformations of the variable helices remain intact. The non-helical conformations change either to helical or partially helical structures. Simulation results of the conserved helices are found to be length dependent, with the shorter helices retaining their conformations and the longer helices breaking into two or more shorter helices. This structural variation is markedly different from the true helix-coil transition in the sense that in this case a given sequence is ambivalent and naturally exists in two different conformations in two different proteins. The amino acid distributions are found to follow completely different patterns for conserved helices and variable helices which may account for the ambivalent nature of the variable and partially ambivalent helices. We report a detailed structural analysis of the ambivalent sequences and find that the amino acid propensities show a marked deviation from their respective values when the sequences are approximately 50% ambivalent. The flanking sequences in both helical and non-helical conformations have distinctly different amino acid preferences and this difference is anisotropic i.e. the N-terminus flanking residues exhibit different amino acid preferences compared to that of the C-terminus flanking sequences. The solvent accessibility results also reveal a similar trend. From this analysis, we conclude that the two flanks of ambivalent sequences possess anisotropic amino acid propensities which may be dictating their preferences for either helical or non-helical conformations.

## Methods

### Database

All *α*-helices of May-2008 release of PDB-select [37] are compiled to create a database from PDB [44] (Protein Data Bank). The database consists of protein chains which have a sequence identity of 25% or less. Only proteins with X-ray crystallographic structures are considered. All protein chains considered in this study have resolution ≤ 3 Å and crystallographic R-factor less than or equal to 0.3. The selected database consists of 2586 non-redundant protein chains from 2466 protein structures. These protein chains may be mapped on to protein chains across the different SCOP classes.

All *α*-helical sequences of the non-redundant database are compared to the SCOP database (release 1.73). SCOP [38] classifies proteins with respect to their structural similarity. Proteins in SCOP are grouped in the hierarchical order of family, superfamily, fold and class, the class being the highest level of hierarchy. In this study, all *α*-helices of the non-redundant database are mapped to identical sequences in the nine SCOP classes viz., (I)All alpha proteins, (II)All beta proteins, (III)Alpha and beta proteins(a+b), (IV)Alpha and beta proteins(a/b), (V)Coiled coiled proteins, (VI)Membrane and cell surface proteins and peptides, (VII)Multi-domain proteins(alpha and beta), (VIII)Peptides and (IX)Small proteins. Two classes namely Designed proteins and Low resolution protein structures are neglected. A structural cutoff of resolution ≤ 3 Å and crystallographic R-factor equal to or less than 0.3 are applied on these protein chains with PISCES server [45]. The final SCOP database consists of 48244 protein chains from 22309 protein structures for comparison.

### Ambivalent helical sequence determination

Secondary structures are annotated residue-wise with the help of DSSP software [41]. According to the widely used definition, H and G are denoted as helical conformation and all other classes (B, E, I, S, T, -) as non-helical [46-48]. Neglecting helices of less than 5 residues long, we have 11592 helices in the non-redundant database. All these helical sequences are mapped into different SCOP classes to find identical sequences. The mapping is done in the following way. For a helix in non-redundant database of *N *residues and a protein chain in SCOP database of *M *residues an *NXM *matrix is created where an element of the matrix, *A*(*i, j*)[*i *= 1 → *N, j *= 1 → *M*], is equal to 1 if *i^th ^*position of the helix and *j^th ^*position of the protein chain have identical residue. Otherwise *A*(*i, j*)[*i *= 1 → *N, j *= 1 → *M*] is equal to 0. Now if an element *A*(*k, l*)[*k*ϵ*i*, *l*ϵ*j*] = 1 and $\sum_{m=0}^{N-1}A\left(k+m,l+m\right)=N$, where *m *is a running variable, then the helix from non-redundant database is said to be mapped in position *l *to *l *+ *N *- 1 of the SCOP protein. Among 11592 helical sequences in the non-redundant database, 6338 occur in SCOP database with varying degree of conformational shift to non-helical conformation. We have binned these helices in a range of 10% conformational shift. For example conserved helices with no conformational shift are allotted 0% bin, conformational shift between 1-10% into 10% bin and so on. Only helices with 100% conformational change are termed as variable helices. It is to be reminded that a given helical sequence obtained from non-redundant database may exhibit different conformational shifts in SCOP, but that helix is placed in the highest percentage bin. For example, if a helical sequence X from the non-redundant database maps into three sequences in SCOP with percentage conformational shifts of 50%, 60% and 70%, then X is binned into 70% bin.

### Molecular dynamics simulation

We performed the molecular dynamics simulations of different helices using AMBER 9 package [49]. The PDB coordinates of the proteins are used and missing hydrogen atoms are added with Leap subroutine. Each protein is solvated in a cubic box with TIP3P water, maintaining a buffering distance of 10 Å between the edge of the box and the protein. The charge of the system is neutralised either with Na+ or Cl- ions. The leaprc.ff99SB force field [50] is used with the periodic boundary conditions. This force field presents a careful re-parametrization of the backbone torsion terms in ff99 and achieves a better balance of four basic secondary structure elements (*PP_II _*, *β*, *α_L _*and *α_R_*). This force field also shows the best agreement with experimental data [51]. Electrostatic interactions are calculated using the PME algorithm [52] with a real space cutoff of 8.0 Å and fourth order spline interpolation. The SHAKE algorithm is used to constrain all bond lengths to their equilibrium distances [53]. Each system is energy minimized twice, first step consists of the energy minimization of the solvent by keeping the protein constrained followed by minimizing the energy of the whole system. A two stage equilibration is performed. The solvated protein is simulated initially at a low temperature of 100 K and the temperature is gradually raised up to 300 K for 10 pico seconds at a constant volume. This is followed by an equilibration for 100 pico seconds at a constant temperature of 300 K and pressure of 1 bar. Constant temperature is maintained through weak coupling to Berendsen temperature bath with coupling constant of 2 pico seconds while constant pressure is maintained through weak coupling to isotropic pressure bath with coupling constant of 1 pico second [54]. Three different production runs of 10 nano seconds are performed for each sequence. All output information are recorded in the production run at an interval of 1 pico second. The time evolution of backbone RMSD with respect to the initial conformation is shown in Figure S1 of Additional file 1 for each sequence. The three plots for each protein show similar pattern with respect to time and the structural deviations are found to be minimal. The divergence of the final set of conformations are measured in terms of the backbone RMSD differences and the average values of RMSD for the three simulations are shown in table T1 of Additional file 1.

## Authors' contributions

P.B. has designed research. N.B. has performed research. P.B. and N.B. have analyzed data and written the manuscript. N.B. and P.B. read and approved the final manuscript.

## Supplementary Material

Additional file 1 **10 nano second simulation results**. This file contains 10 nano second molecular dynamics simulation results of variable and conserved helices. The figures presented in this file depict the initial and the final structure of the proteins during molecular dynamics simulation. The time evolution of secondary structures for variable and conserved helices are also provided here. Click here for file

Additional file 2 **1 nano second simulation results**. This file contains 1 nano second molecular dynamics simulation results of variable and conserved helices. Variable helices are simulated by different protocols viz., simulation of the target chain, simulation of the target chain by constraining all other chains, simulation of the whole protein. RMSD curves for the helices which follow a large deviation from the initial conformations are also provided. Click here for file
