# Supplementary material for: Statistical analysis and molecular dynamics simulations of ambivalent α -helices
Source: BMC Bioinformatics. 2010 Oct 18;11:519. doi: 10.1186/1471-2105-11-519 (PMC2973962; doi:10.1186/1471-2105-11-519)

Figure.S1. Time evolution of backbone RMSD for three separate production runs for each of the three protein chains 1H4LD, 1UNGE and 1BH8B. The detail results of the first simulation for each of them are presented in the main manuscript.

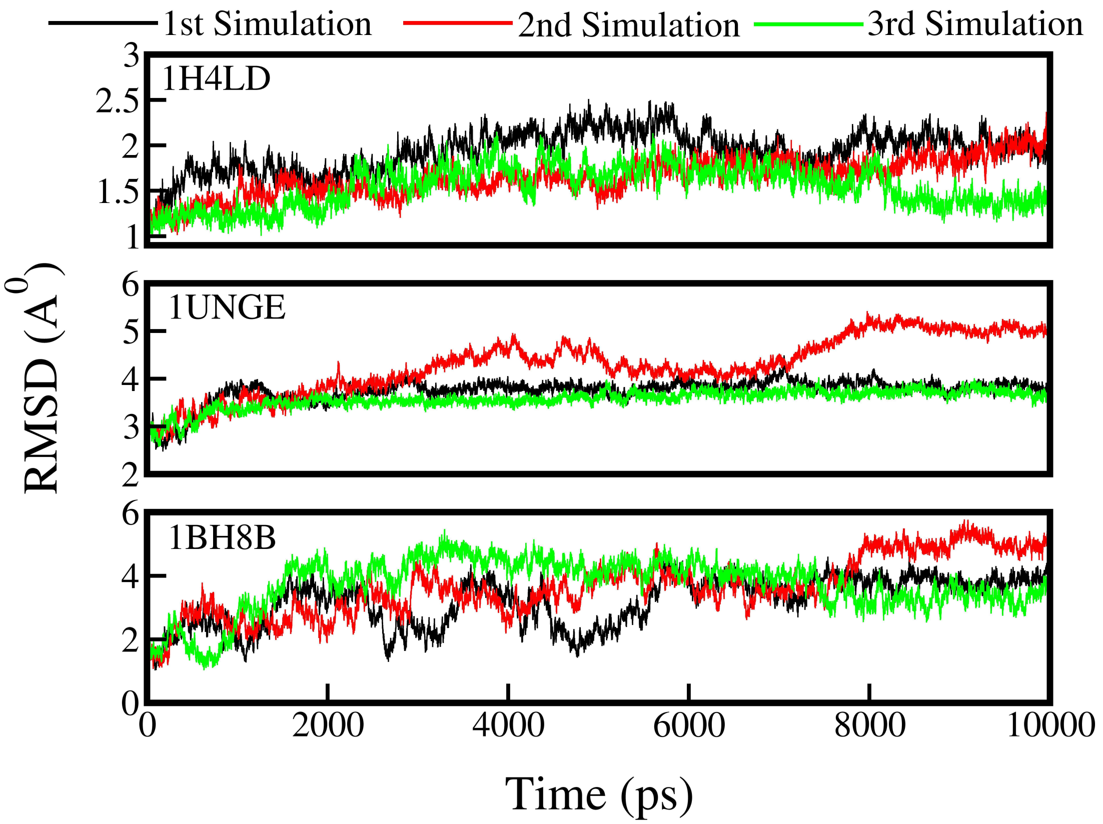

Table S1. Average backbone RMSD between the final conformations of each protein in three production run given in figure S1.

**Protein Chain**	**RMSD (A°)**				
	Whole Protein	1st Helix/non-helix	2nd Helix/non-helix	3rd Helix/non-helix	4th Helix/non-helix
1H4LD	1.419	0.561	-	-	-
1UNGE	2.747	2.371	-	-	-
1BH8B	2.751	1.062	1.533	2.325	0.303

Figure.S2. Time evolution of secondary structures for four conserved helices in 1BH8B.

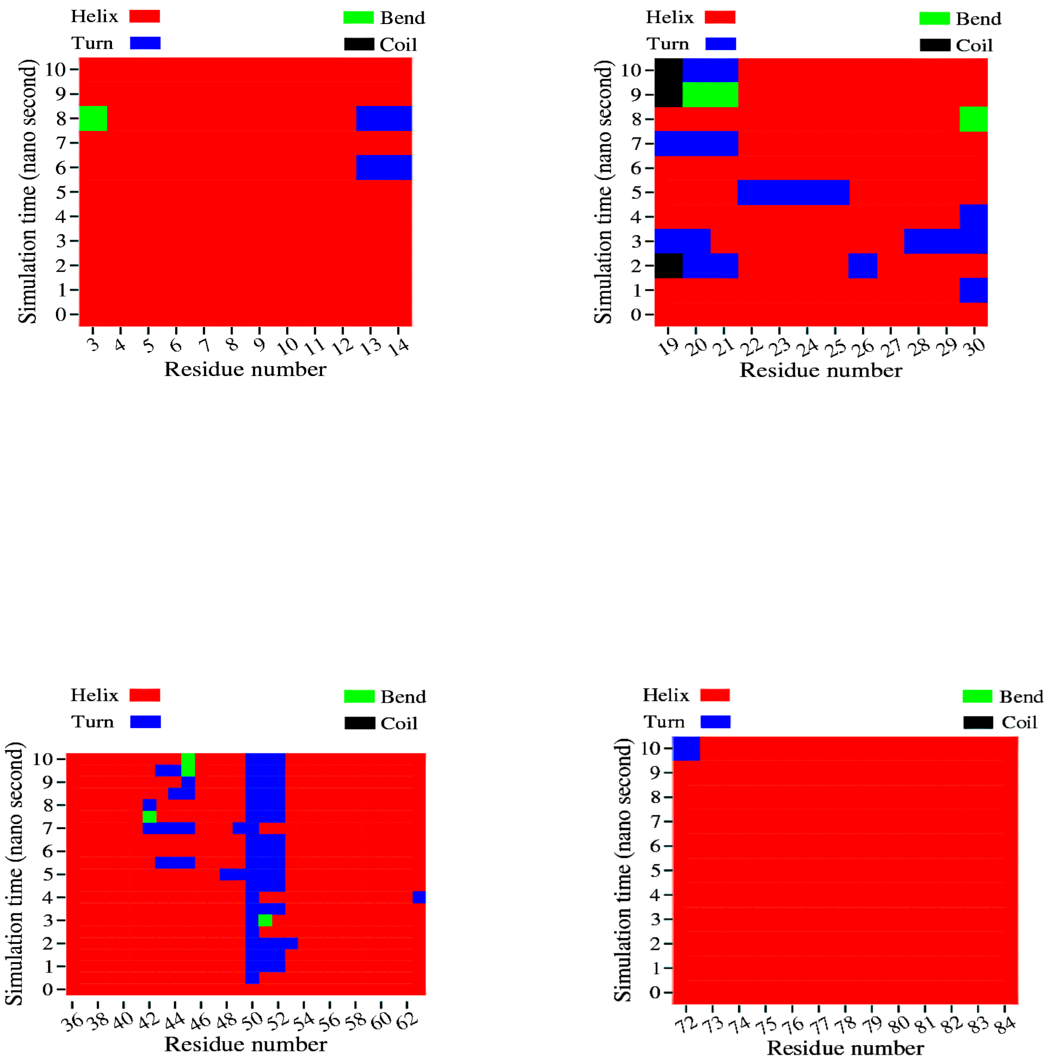

Figure.S3. Initial and final structures of the protein chain 1IJDB containing a conserved helix at position 6-36 (SEQAEELHKHVIDGTRVFLVIAAIAHFLAFT) after 10 nano second simulation. Below the time evolution of secondary structures in the helical sequences is provided.

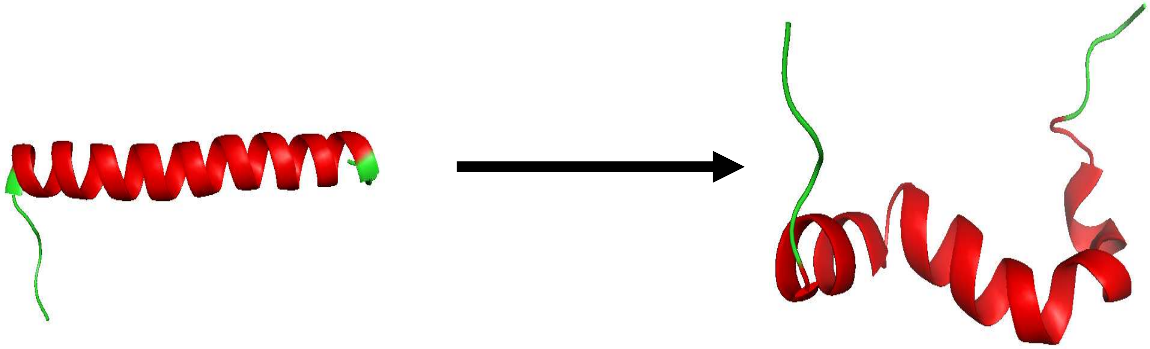

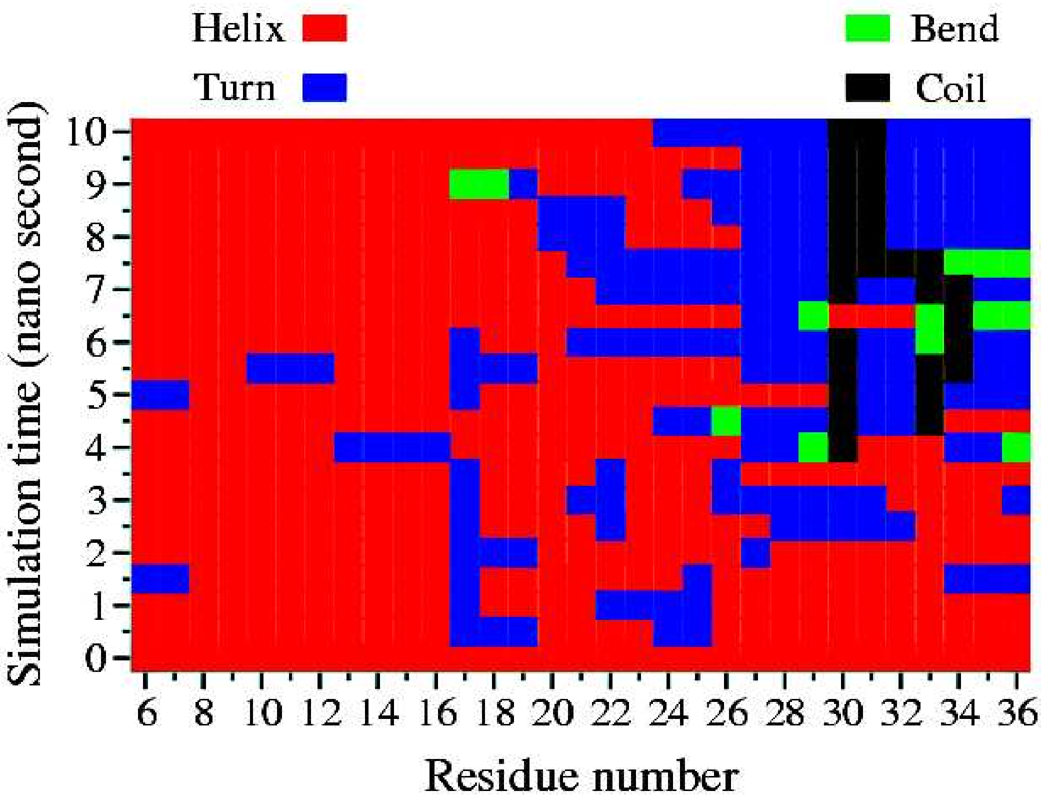

Figure.S4. Initial and final structures of protein chain 1M93A containing a conserved helix at position 20-33 (PPSISSVLTILYYG) after 10 nano second simulation. Below time evolution of secondary structures in the helical sequence is provided.

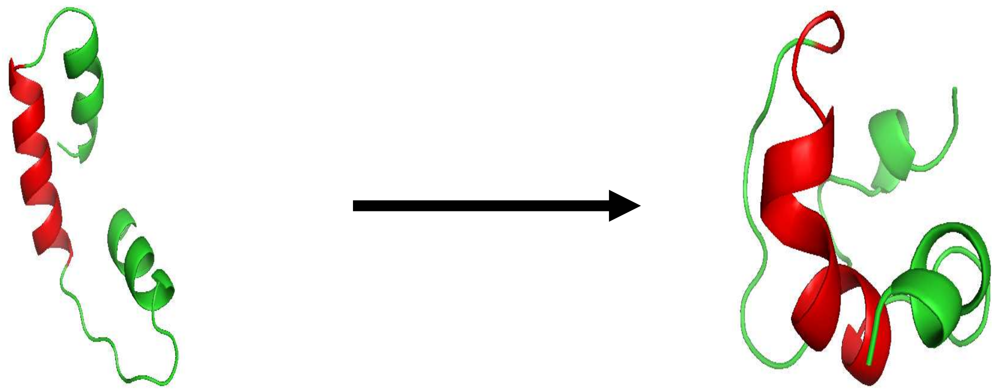

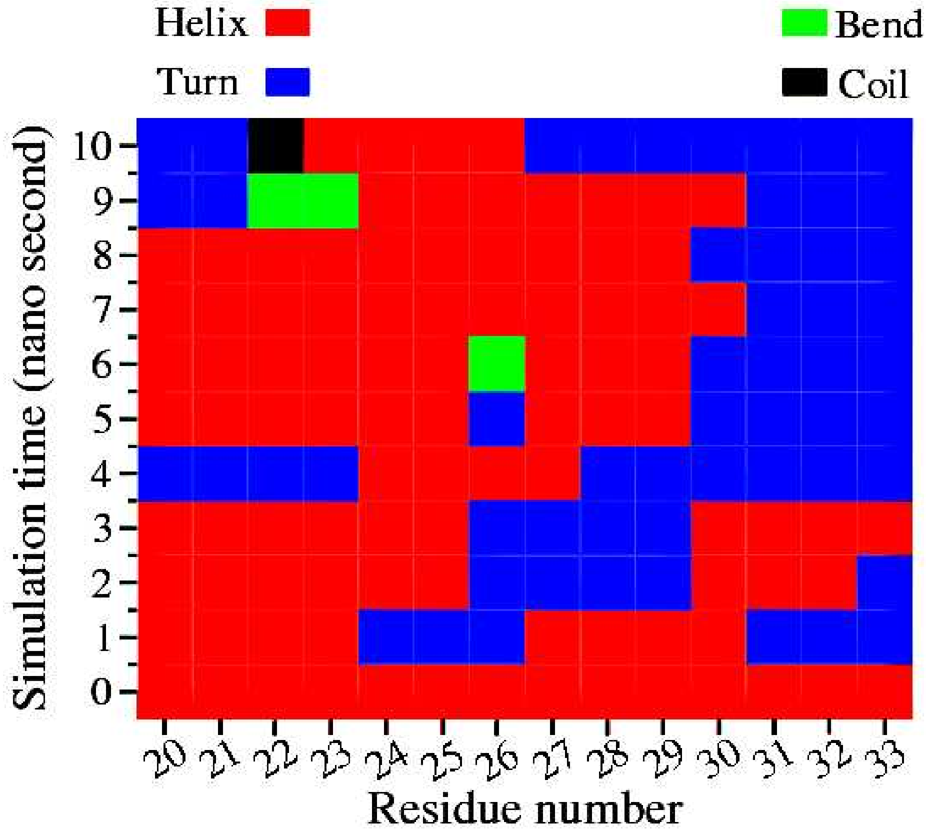

Figure.S5. Initial and final structures of protein chain 1KA8A containing two conserved helices at positions 37-47 (LYHAYLAYMEA) and 56-69 (LKMFGLGLPVMLKE) after 10 nano second simulations. Below time evolutions of secondary structures in the helical sequences are provided.

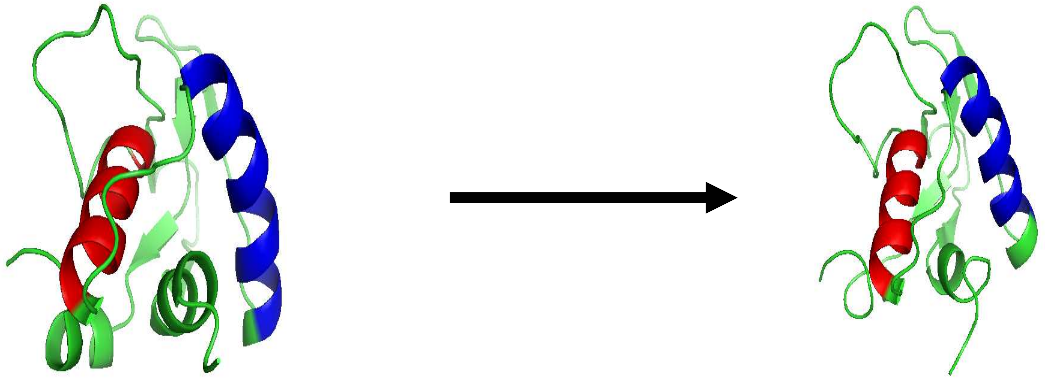

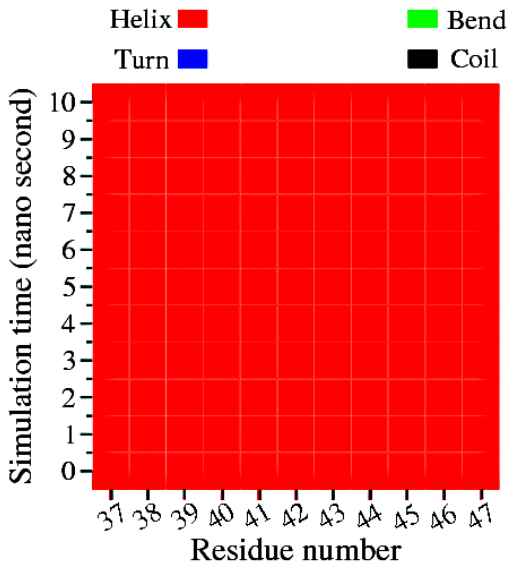

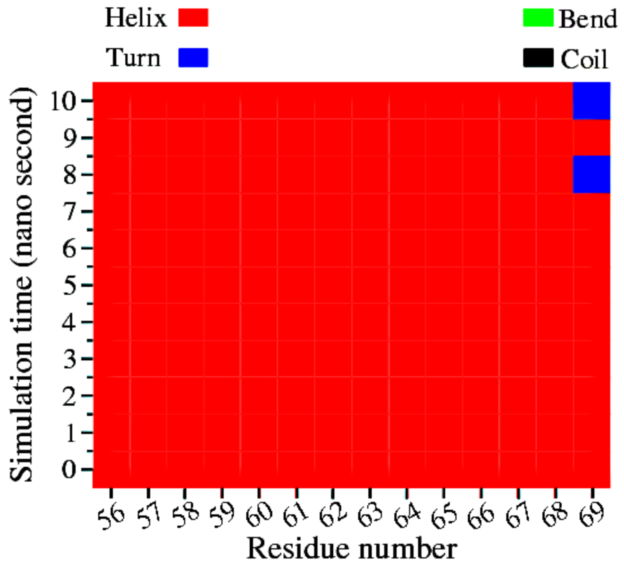

Figure.S6. Initial and final structures of protein chain 1AHOA containing a conserved helix at position 19-28 (NAYCNEECTK) after 10 nao second simulations. Below time evolution of secondary structures in the helical sequence is provided.

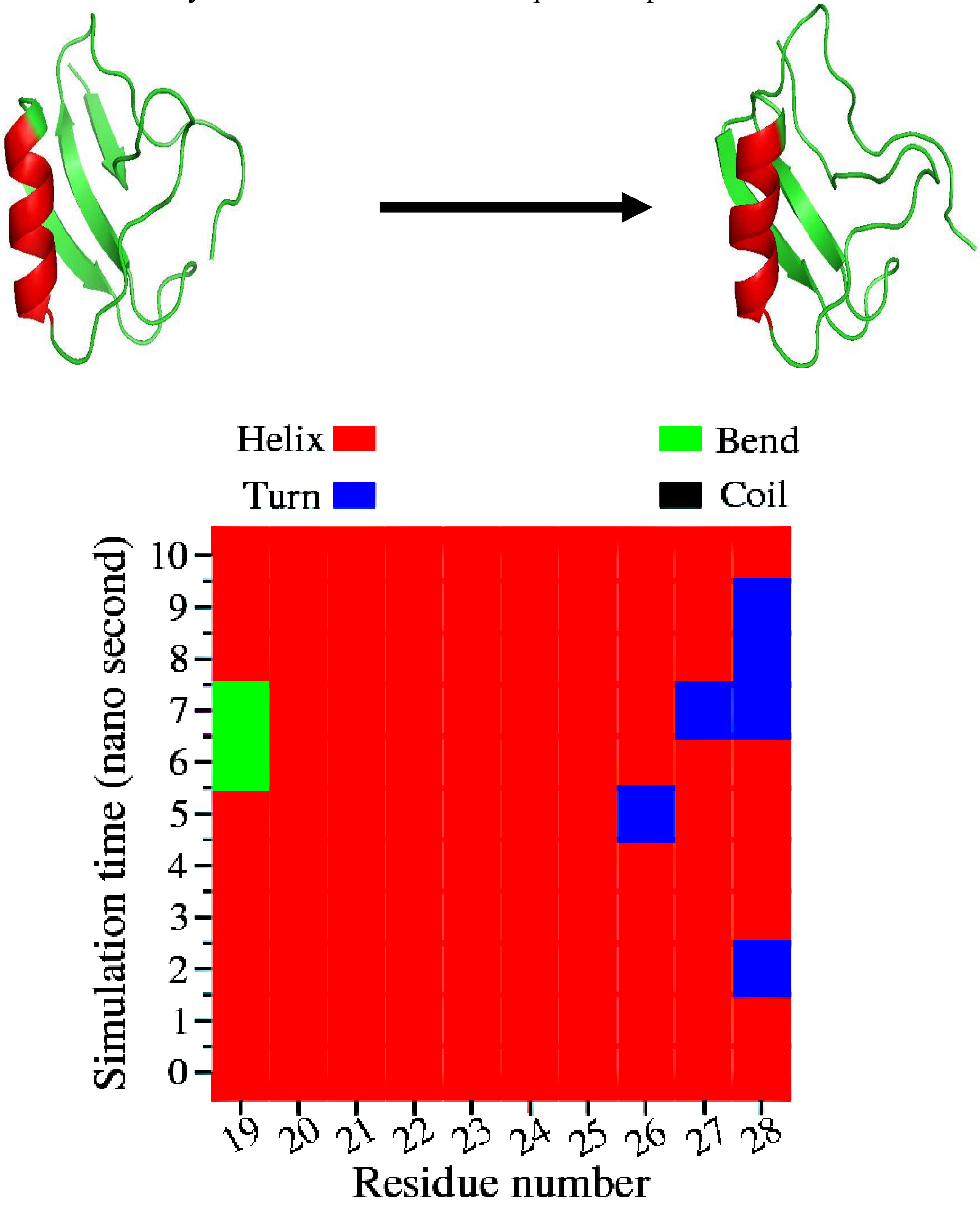

Figure.S7. Initial and final structures of protein sequence 1T9IB containing a helical conformation of variable helical sequence at position 144-152 (SETVRAVLD) after 10 nano second simulations. Below time evolution of secondary structures in the helical sequence is provided.

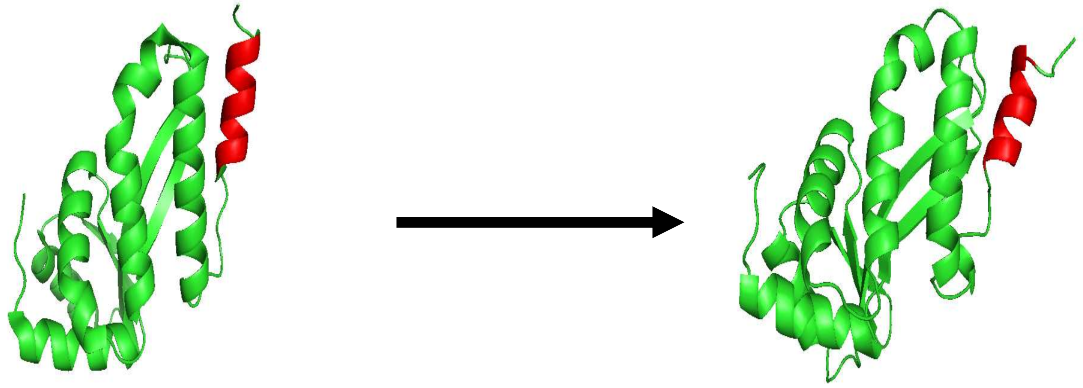

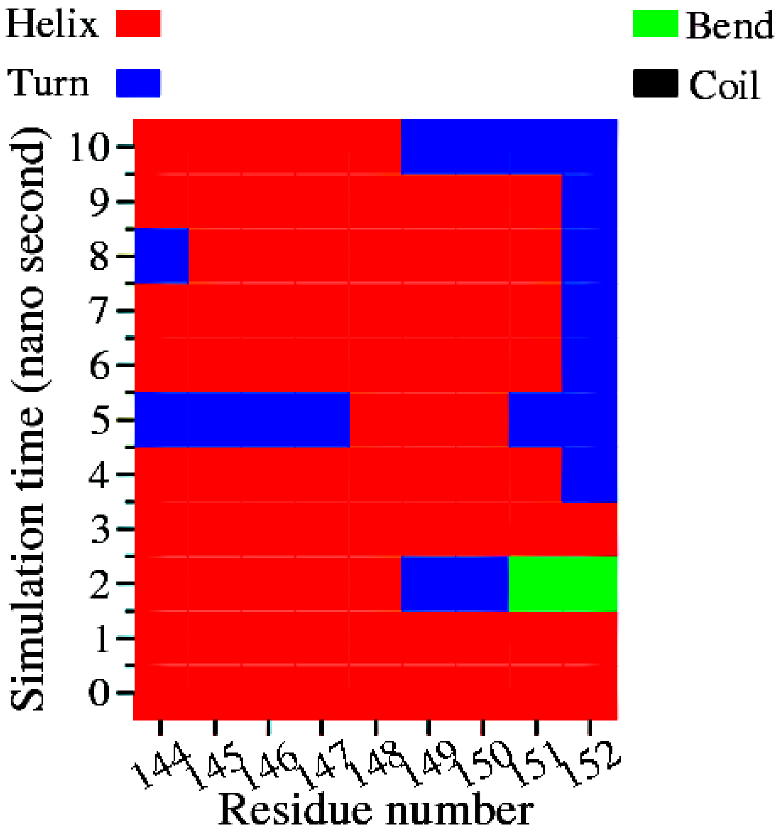

Figure.S8. Initial and final structures of protein chain 1U0DA containing non-helical conformation mapped by helical sequence of 1T9IB after 10 nano second simulations. Below time evolution of secondary structures in non-helical sequence is provided.

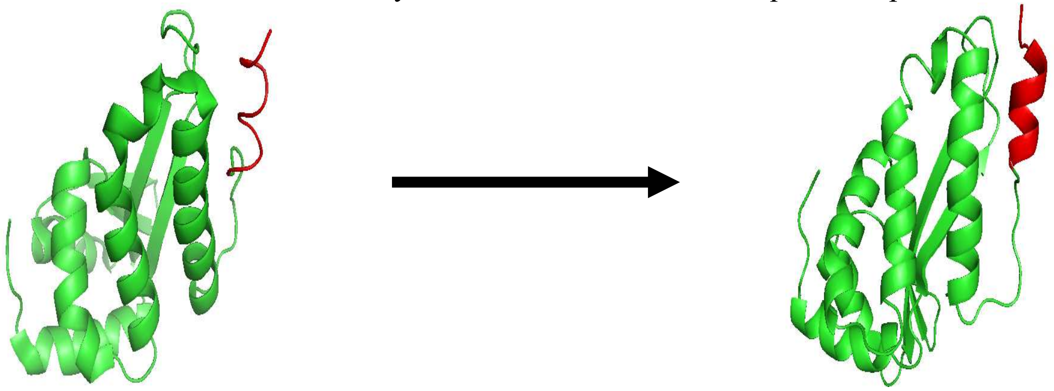

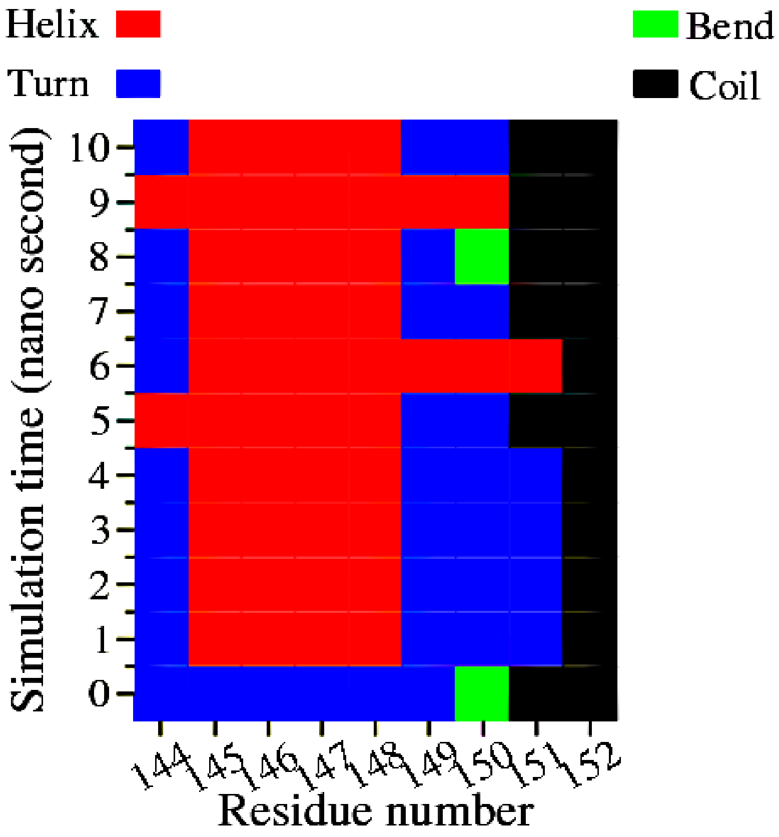

Figure.S9. Initial and final structures of protein chain 1NQJB containing helical conformation of a partially ambivalent helical sequence at position 4-15 (LKEKENNDSSDK) after 10 nano second simulation. Below time evolution of secondary structures in the helical sequence is provided

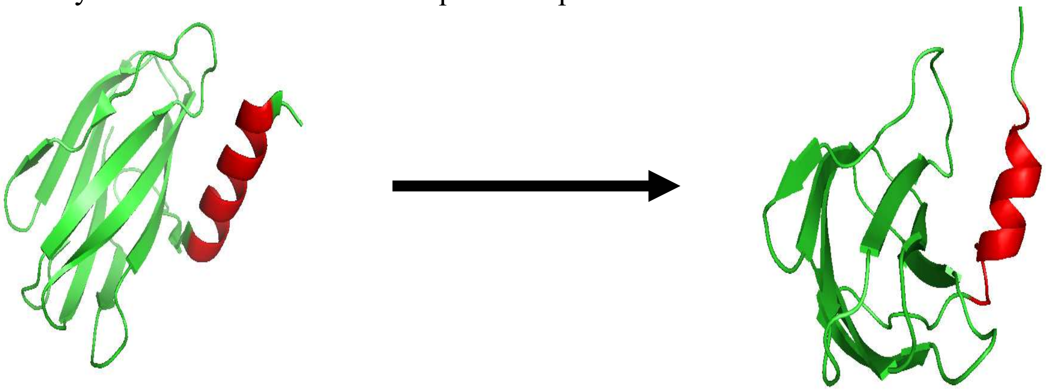

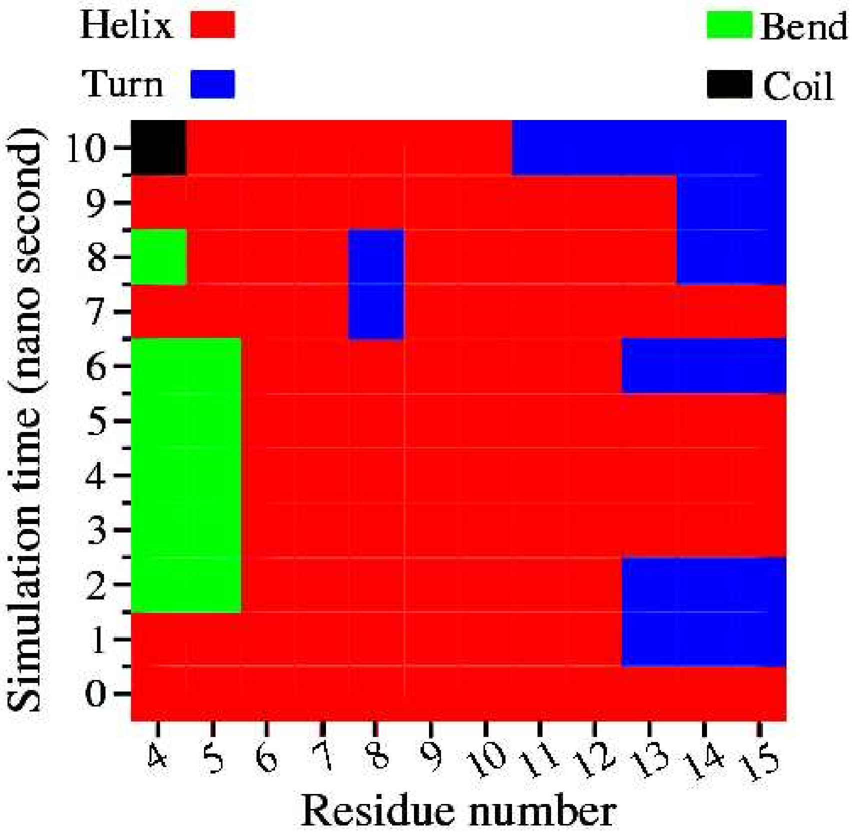

Figure.S10. Initial and final of protein chain 1NQDB containing the partial non-helical conformation mapped by helical sequence of 1NQJB after 10 nano second simulation. Below time evolution of secondary structure in the partial non-helical sequence is provided.

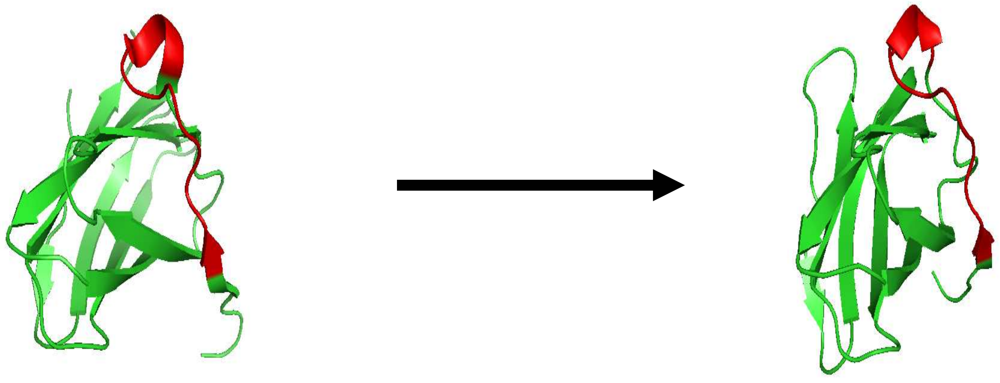

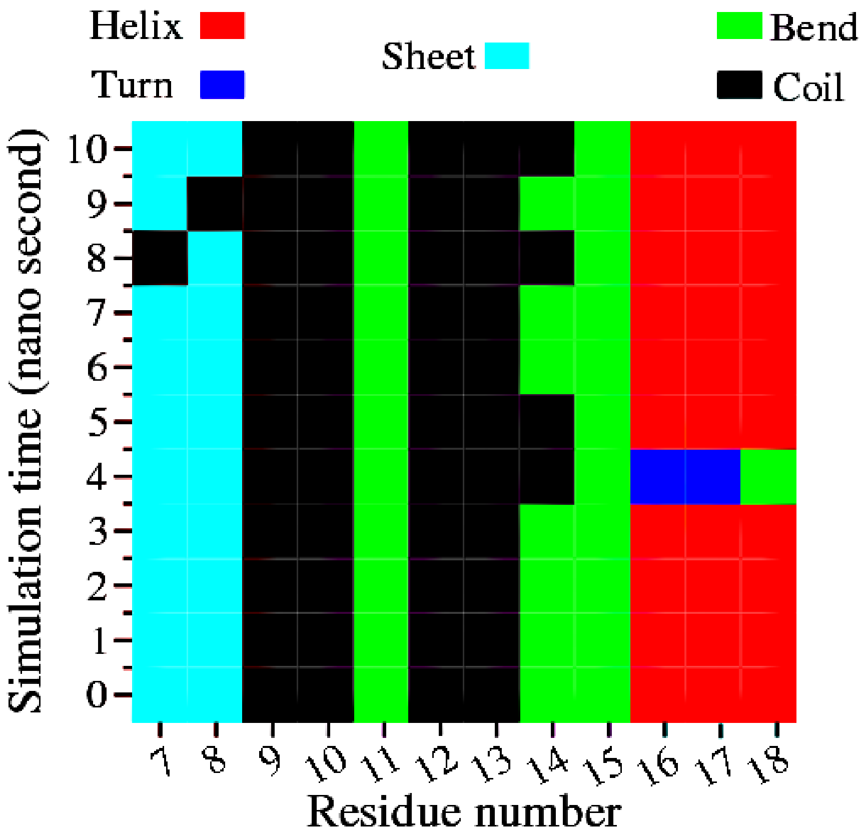

Supplement: Additional file 1 — 10 nano second simulation results. This file contains 10 nano second molecular dynamics simulation results of variable and conserved helices. The figures presented in this file depict the initial and the final structure of the proteins during molecular dynamics simulation. The time evolution of secondary structures for variable and conserved helices are also provided here. [file 1471-2105-11-519-S1.PDF]
