# Supplementary material for: Statistical analysis and molecular dynamics simulations of ambivalent α -helices
Source: BMC Bioinformatics. 2010 Oct 18;11:519. doi: 10.1186/1471-2105-11-519 (PMC2973962; doi:10.1186/1471-2105-11-519)
Supplement: Additional file 2 — 1 nano second simulation results. This file contains 1 nano second molecular dynamics simulation results of variable and conserved helices. Variable helices are simulated by different protocols viz., simulation of the target chain, simulation of the target chain by constraining all other chains, simulation of the whole protein. RMSD curves for the helices which follow a large deviation from the initial conformations are also provided. [file 1471-2105-11-519-S2.PDF]

<u>Variable helical sequences</u>

1. MD of only target chain

i)

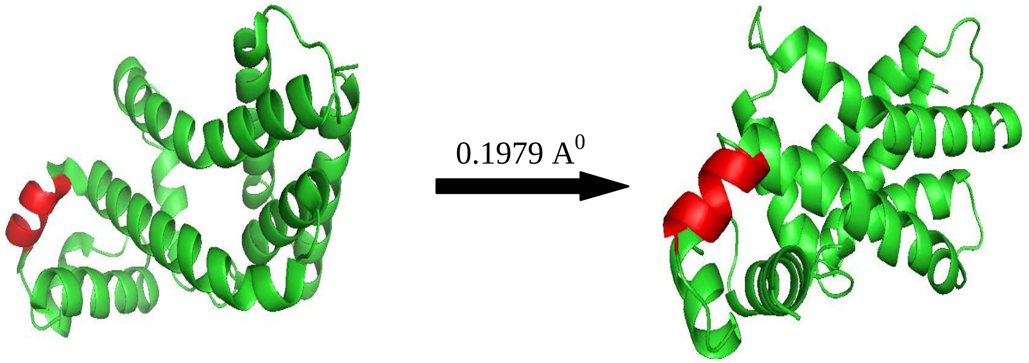

1JUMA

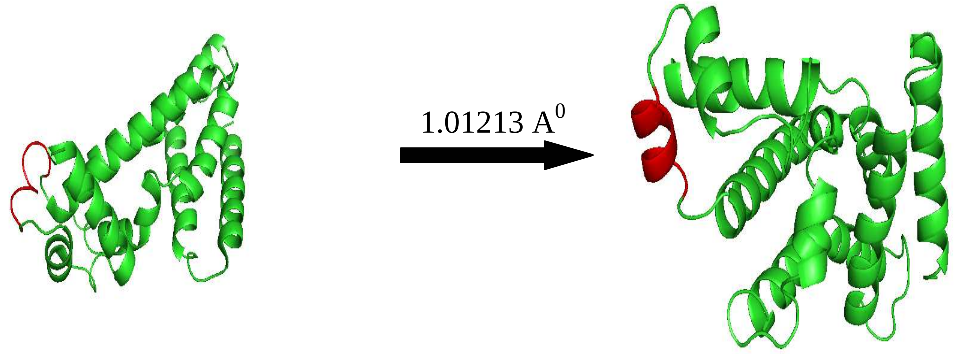

1RPWA

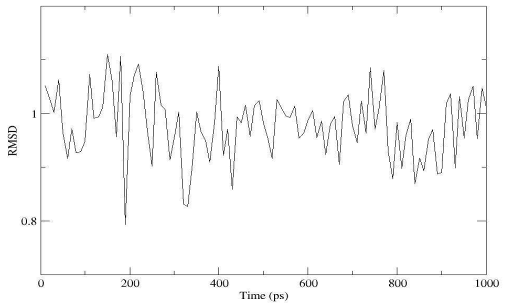

Helical segment 36-43 of 1JUMA (KGNLYYHF) map into SCOP protein 1RPWA at 36-43. Both of them belong to the SCOP class All Alpha Protein. The helical segment in 1JUMA remains helical while non helical segment changes to helix. RMSD curve of non helical segment is provided for the production run.

ii)

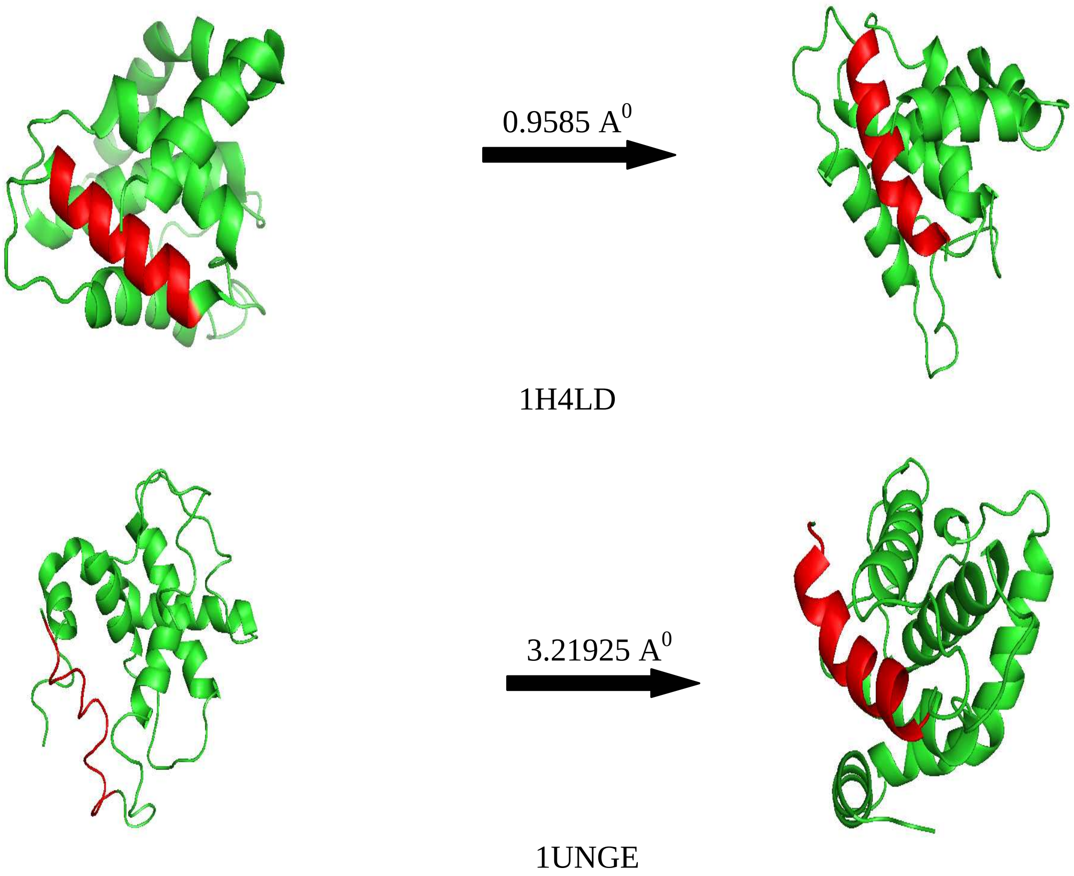

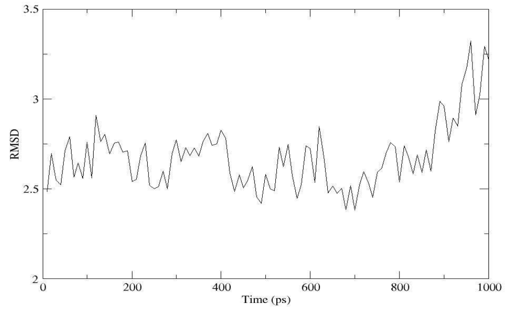

Helical segment 148-162 of 1H4LD (TSELLRCLGEFLCRR) map into SCOP protein 1UNGE at 148-162. Both of them belong to the SCOP class All Alpha Protein. The helical segment in 1H4LD remains helical while non helical segment changes to helix. RMSD curve of non helical segment is provided for the production run.

iii)

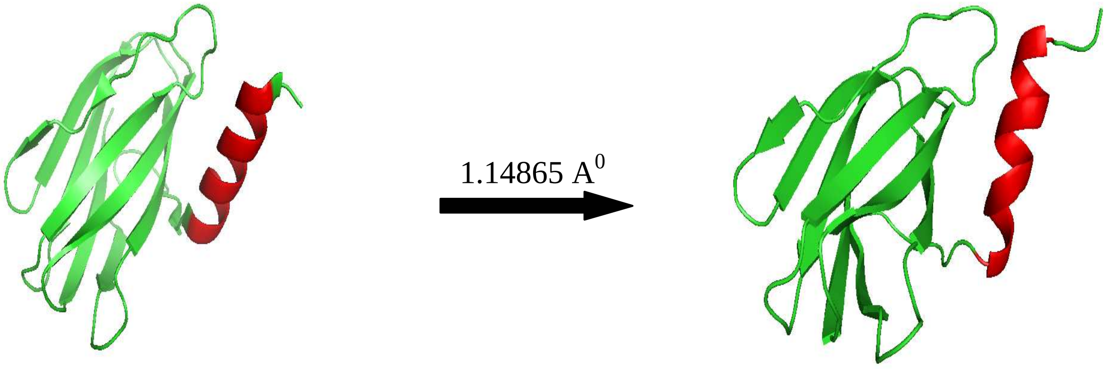

1NQJB

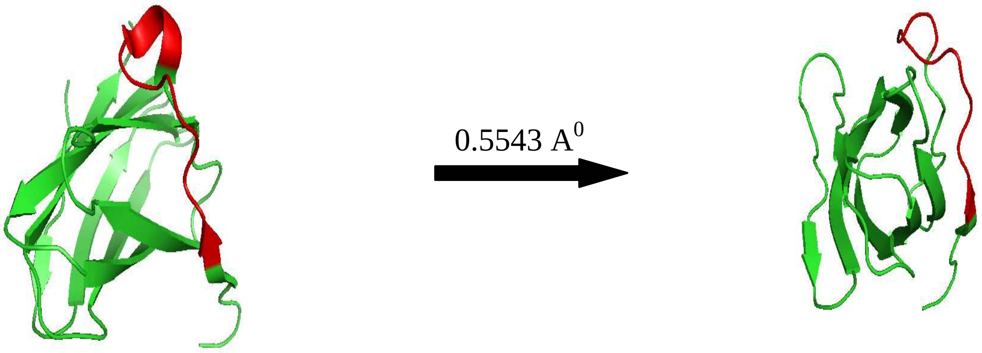

1NQDB

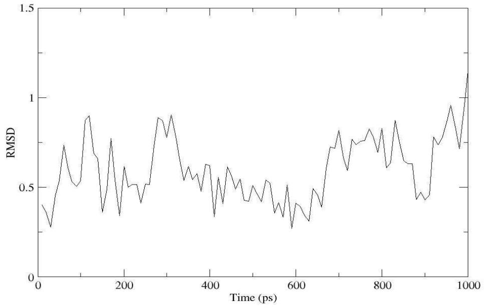

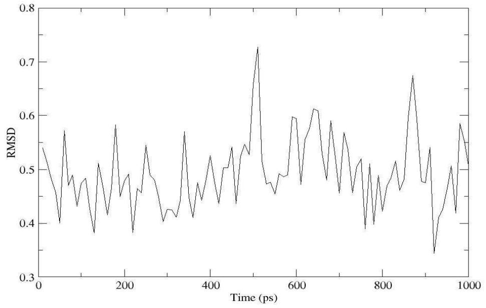

Segment 897-908 (LKEKENNDSSDK) of 1NQJB from non redundant database is helical sequence which map in 1NQDB at 897-908 as non helical conformation. Both of them belong to All Beta Protein class of SCOP. After 1 nano second MD simulation the helical conformation transforms into a distorted helix while the non helix remain as it is with minimal structural deviation. RMSD curves of the segments are shown above (LHS for 1NQJB and RHS for 1NQDB)

iv)

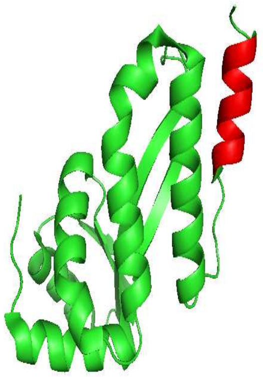

0.23003 A^0

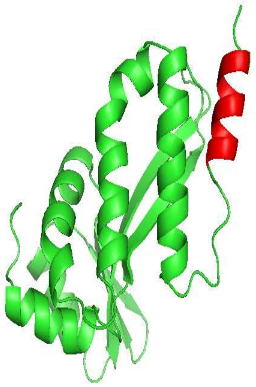

1T9IB

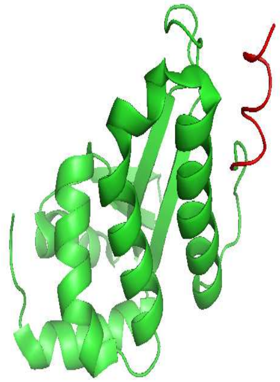

1.61546 A^0

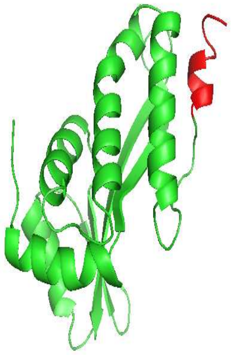

1U0DA

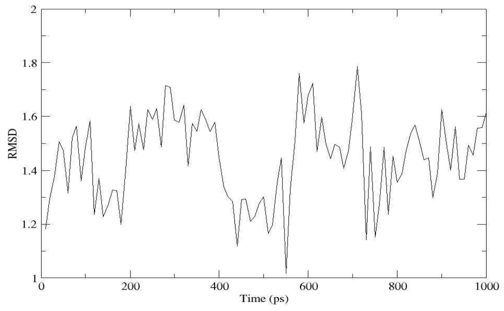

Both 1T9IB and 1U0DA belong to Alpha & Beta Proteins (A+B) class of SCOP. Segment 445-453 (SETVRAVLD) in non redundant protein 1T9IB is helical while the same segment at 145-153 in 1U0DA is non helical. After 1 nano second of MD the helical sequence remains helix with little deviation while the non helical changes to partially helical conformation. The RMSD at production run for non helical segment is given above.

v)

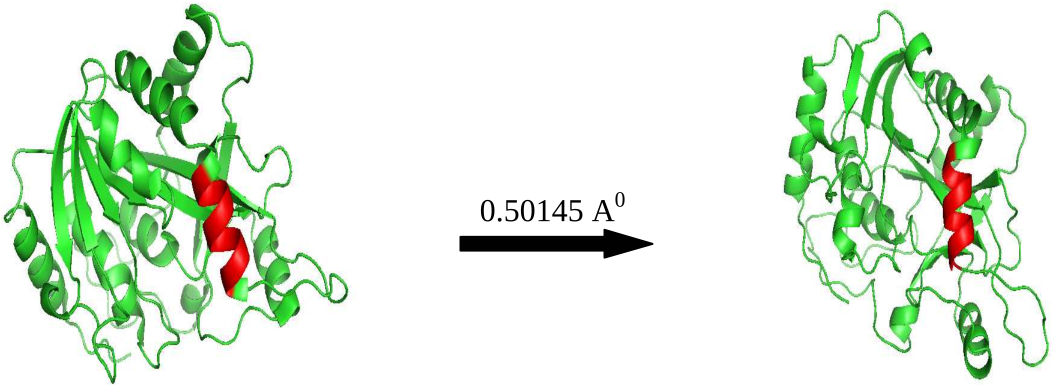

2Q7OE

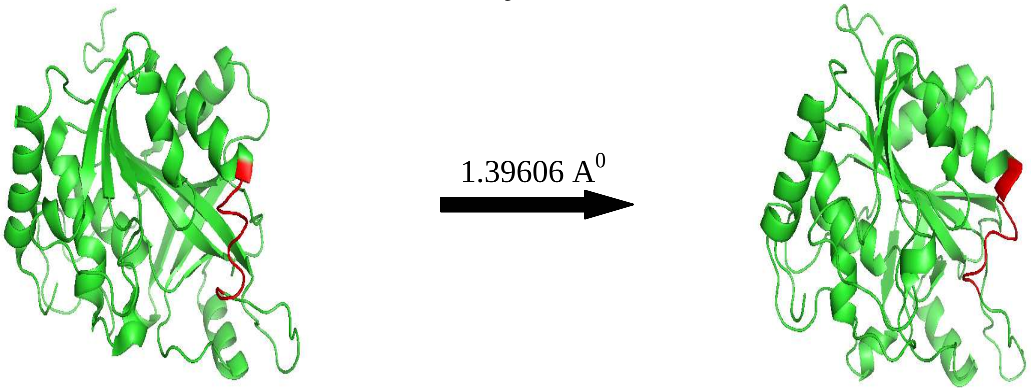

1RCTE

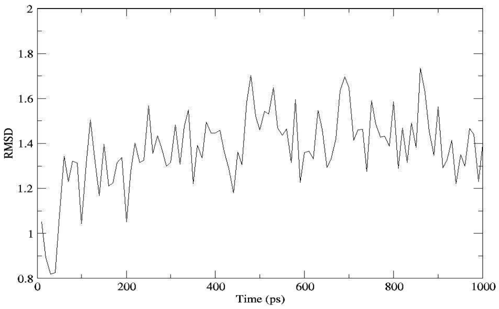

Helical segment 257-265 (HEEVLAAGK) of 2Q7OE map into segment 256-264 of 1RCTE which is non-helical. 2Q7OE is not classified b SCOP while 1RCTE belongs to Alpha and Beta proteins class of SCOP. After 1 nano second of MD both the helical and non-helical conformation remain intact. RMSD curve of non helical segment is provided for the production run.

2. MD by constraining chains excluding the target

i)

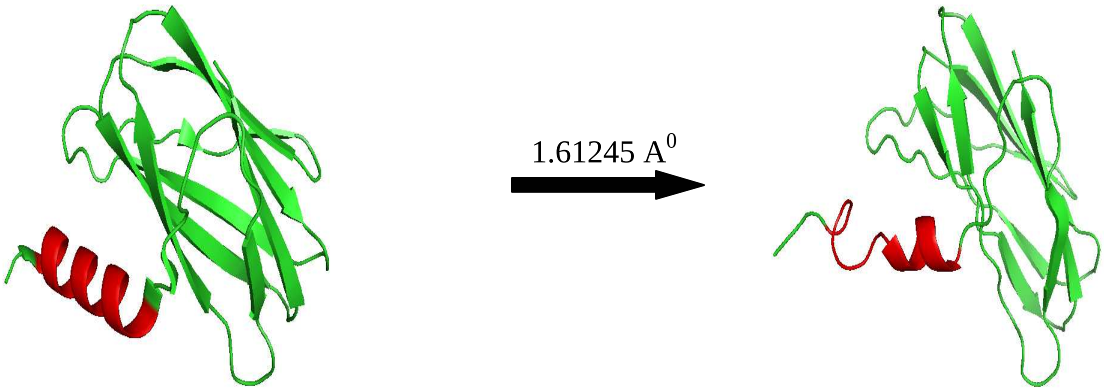

1NQJB

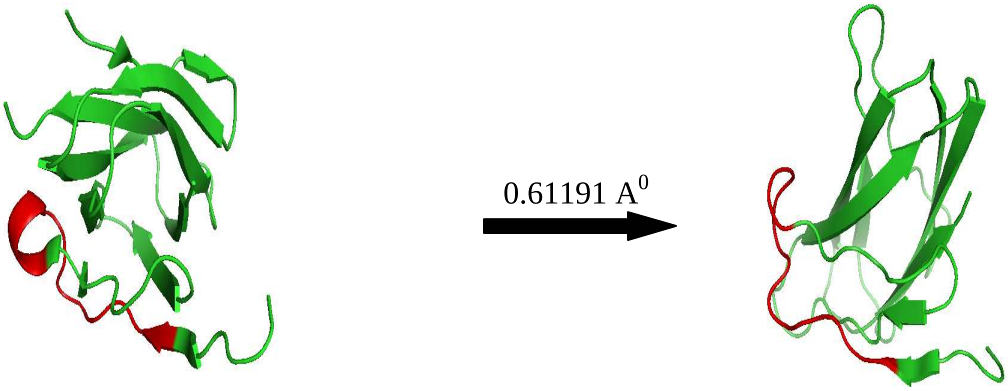

1NQDB

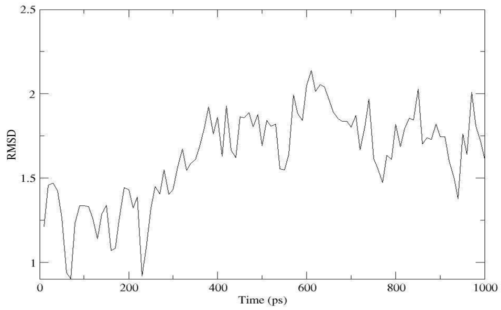

MD on 1NQJB and 1NQDB are again repeated by running the simulation on the whole protein. For both of them the other chain, chain A in both cases, are constrained while running MD. The outcome remains same for 1NQDB i.e. the non helical conformation remain non helical with little distortion. While the helical conformation of 1NQJB change to partial helical segment.

3. MD of total protein

i)

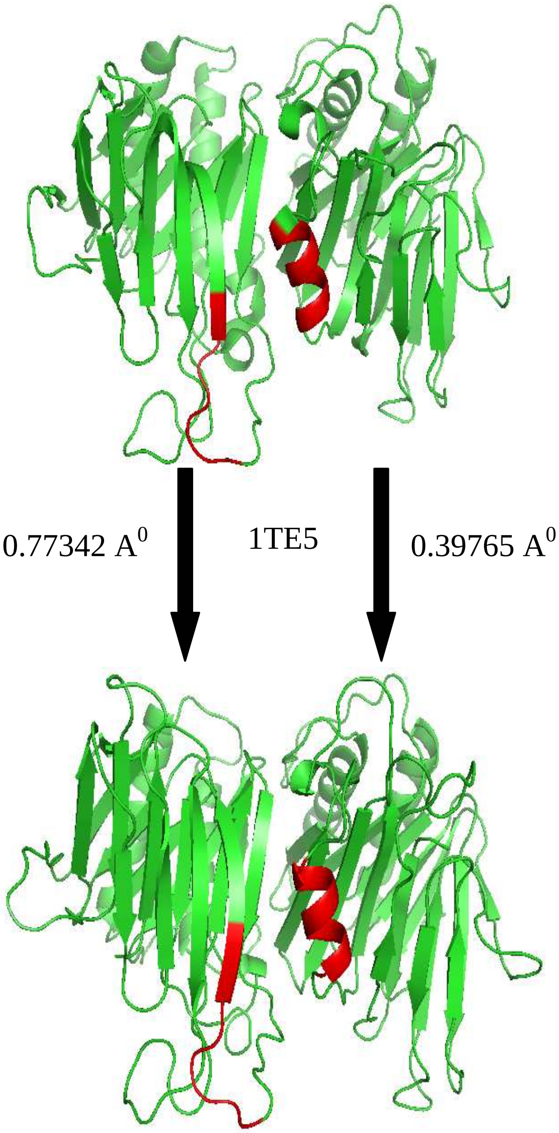

For protein 1TE5 the segment (EVARLVQR) 60-67 in A chain is helical, it is non helical in 60-67 in B chain. 1TE5 belongs to Alpha & Beta Proteins class of SCOP. MD results show that the ambivalent segment is quite stable in both the helical and non helical conformations and the RMSD between the initial and final structures of the segments are very low.

ii)

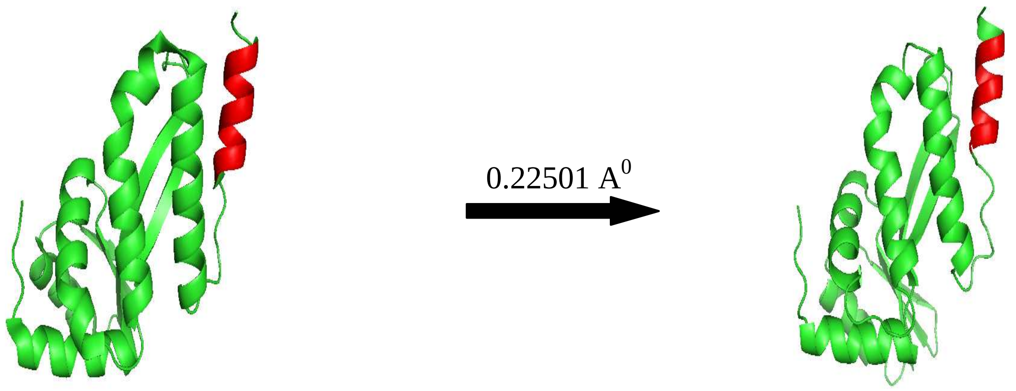

1T9IB

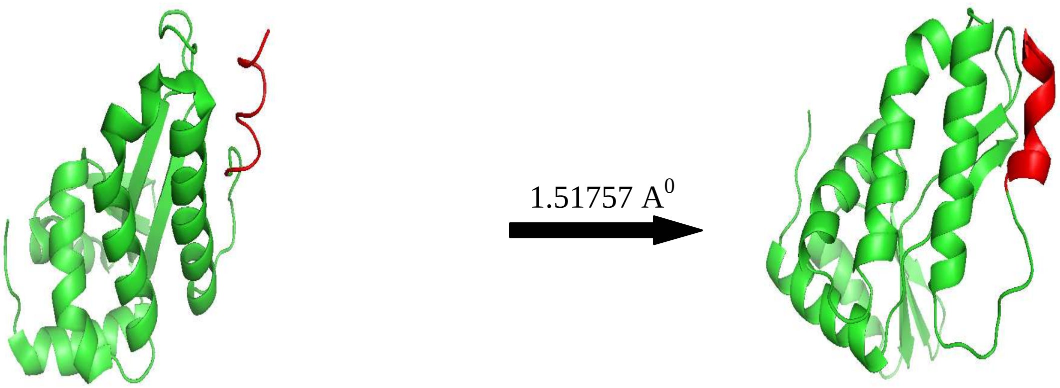

1U0DA

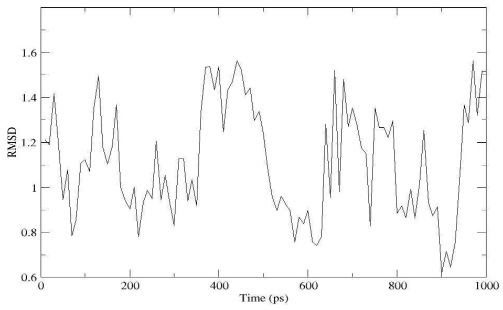

The MD on 1T9IB and 1U0DA are repeated by simulating the whole protein. The result for 1T9IB is similar to previous observations. In case of the non helical conformation in 1U0DA it shifts to a distorted helix instead of partial helix after simulation. The RMSD curve for the non helical segment of 1U0DA is shown above.

<u>Conserved helical sequences</u>

i)

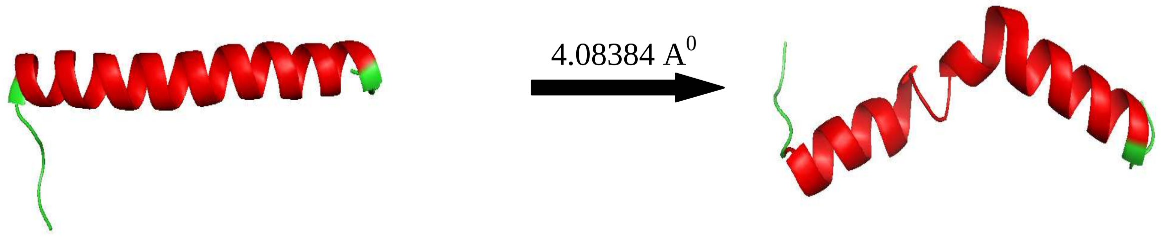

1IJDB

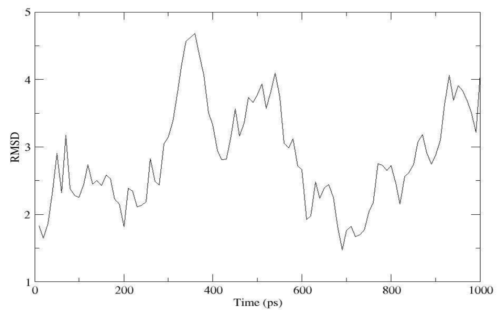

Segment 6-36 (SEQAEELHKHVIDGTRVFLVIAAIAHFLAFT) of protein chain 1IJDB belonging to Membrane and Cell Surface Proteins and Peptides class of SCOP is a conserved helical sequence in our data base. Few residues at the middle of this helix changes to non helical conformations leading to a high RMSD between the initial and final structure of this segment after MD.

ii)

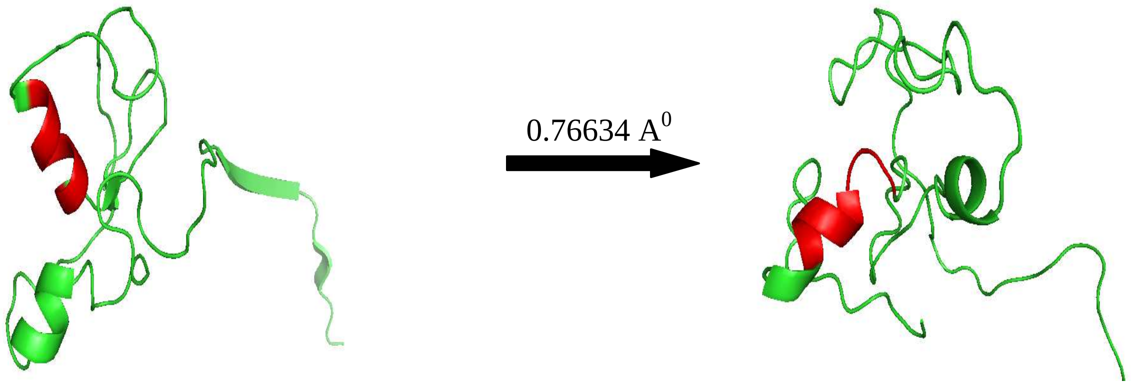

1LDJB

Protein chain 1LDJB belongs to Small Proteins class of SCOP. Segment 82-88 (HCISRWL) of this chain belongs to conserved helical sequence in our data base. The segment retains its helical conformation after MD run and the RMSD between initial and final structure is low.

iii)

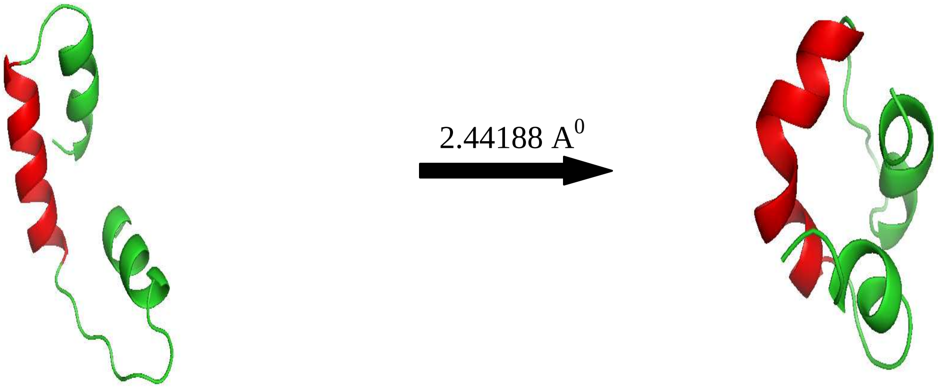

1M93A

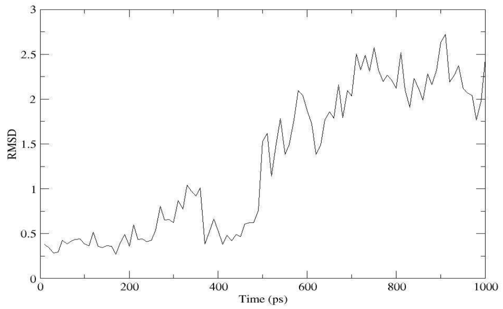

Segment 20-33 (PPSISSVLTILYYG) of protein chain 1M93A is a rigid helical sequence. The protein chain belongs to Multi Domain Protein class of SCOP. After MD simulation the segment shifts to a distorted helical conformation. Hence our RMSD plot of the segment in production run shows a high RMSD between initial and final structure.

iv)

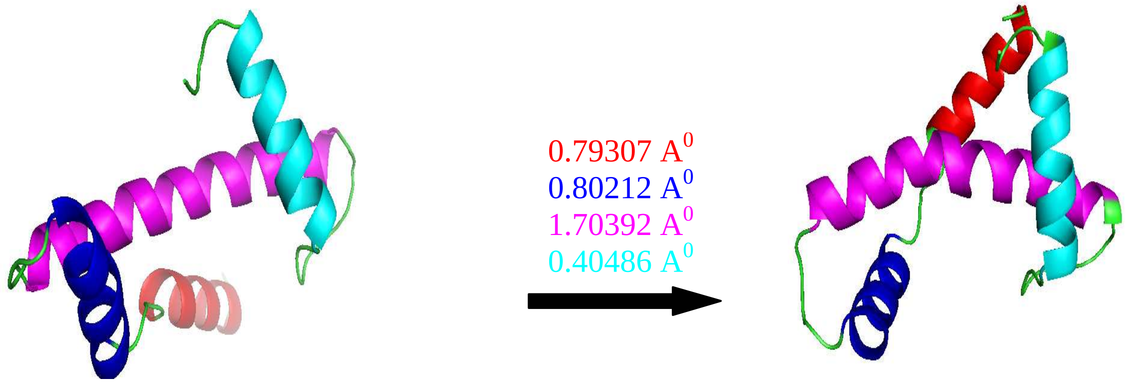

1BH8B

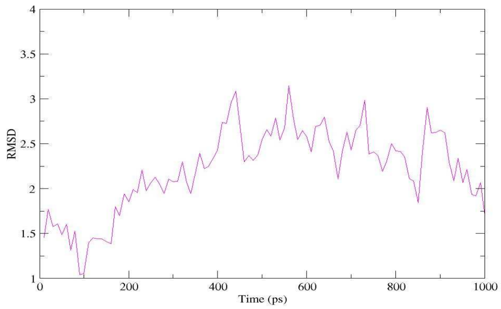

Protein chain 1BH8B belongs to All Alpha Proteins class of SCOP. It contains four conserved helical sequences: segment 115-126 (EEQLNRYEMYRR), segment 131-142 (KAAIKRLIQSIT), segment 148-175 (QNVVIAMSGISKVFVGEVVEEALDVCEK) and segment 184-196 (PKHMREAVRRLKS). After MD simulation only the third helical sequence, segment 148-175, have some distortion in the mid region. This results into slightly high RMSD between initial and final structure of this segment.

v)

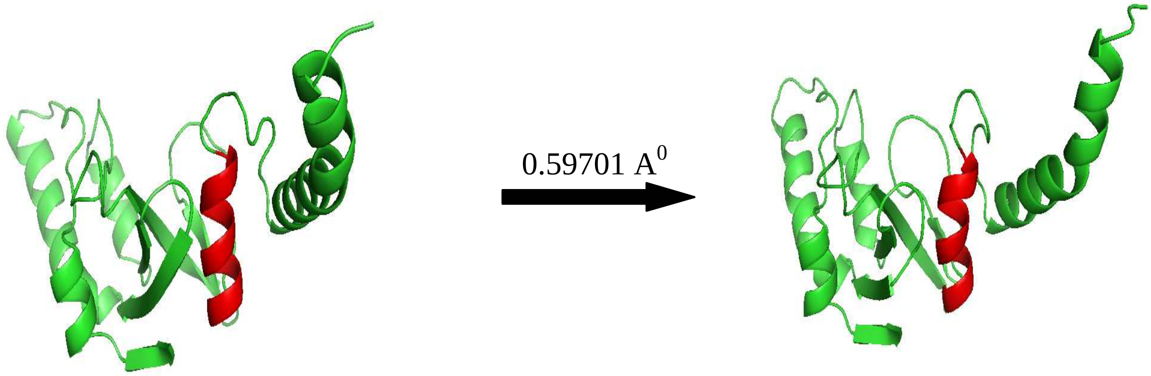

1H56A

Segment 36-46 (GGKLLQVLLIT) of protein chain 1H56A belongs to conserved helical sequence in our data base. The protein chain is from Alpha & Beta Proteins (A/B) of SCOP. There is minimal deviation in the helical conformation of this segment after MD simulation.

vi)

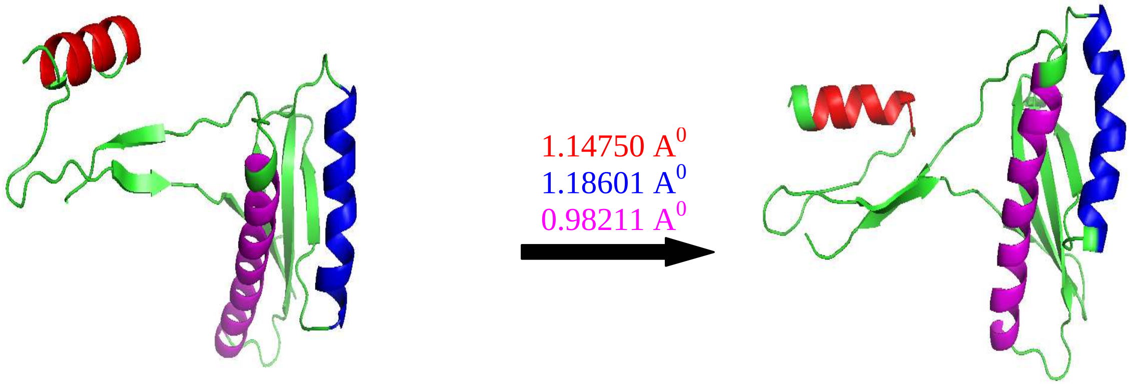

1K3EB

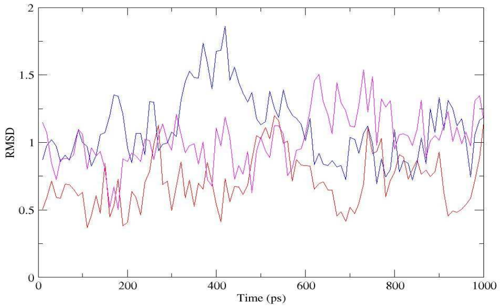

The protein chain 1K3EB, belonging to Alpha & Beta Proteins (A+B), consists of three conserved helical sequences: segment 6-16 (ELLLEKFAEKI), segment 63-78 (SNFALEILNANLWFAE) and segment 106-128 (PEKLENEIEVVVKSMENLYLVLH). After MD simulation all three helices acquire slight distortion which results into slightly higher RMSD of initial and final structure of these helices.

vii)

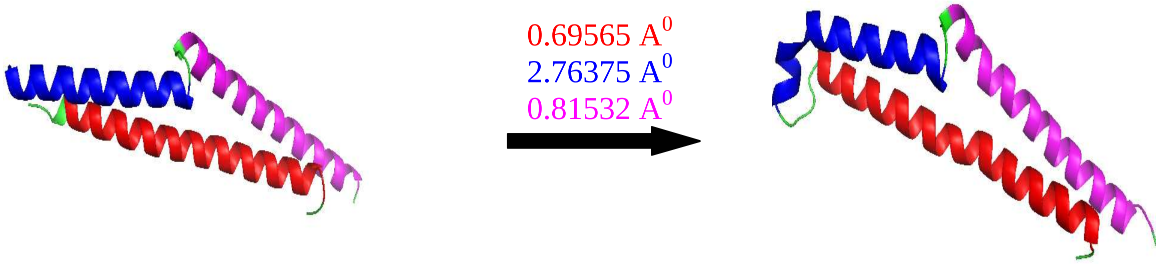

1M5IA

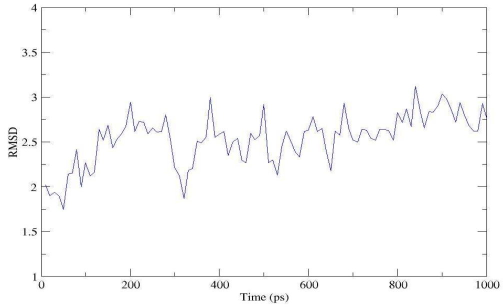

Protein chain 1M5IA belongs to Coiled Coil Proteins class of SCOP. It has three conserved helical sequences: segment 132-169 (GYLEELEKERSLLLADLDKEEKEKDWYYAQLQNLTKRI), segment 180-204 (LQTDMTRRQLEYEARQIRVAMEEQL) and segment 208-238 (QDMEKRAQRRIARIQQIEKDILRIRQLLQSQ). The second helical sequence acquires a higher RMSD between initial and final structure due distortion of the helix at the mid region after MD simulation.

viii)

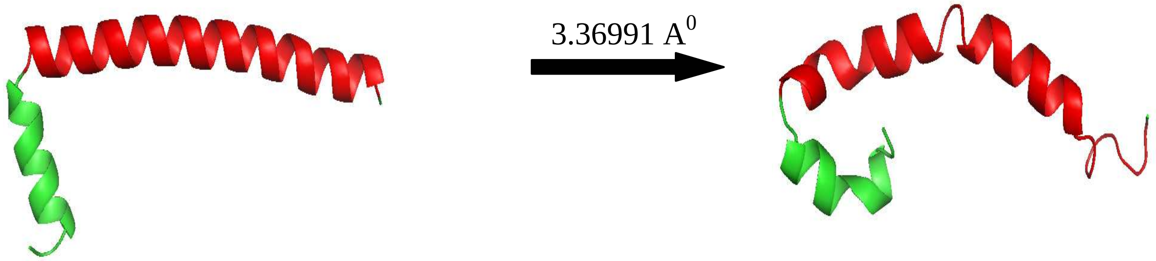

1A92A

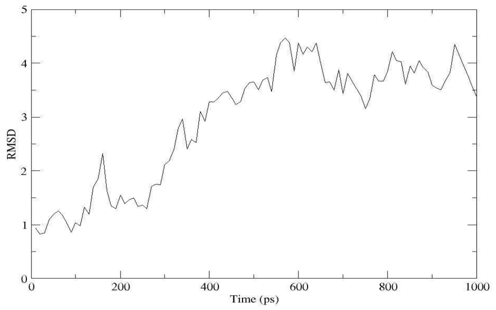

Segment 13-47 (REDILEQWVSGRKKLEELERDLRKLKKKIKKLEED) of protein chain 1A92A belongs to conserved helical sequence. The protein chain is from Coiled Coil Proteins class of SCOP. Few mid residues of the helix transit to non helical conformations after MD simulation. This leads to a high RMSD between initial and final structure of this helical segment.

ix)

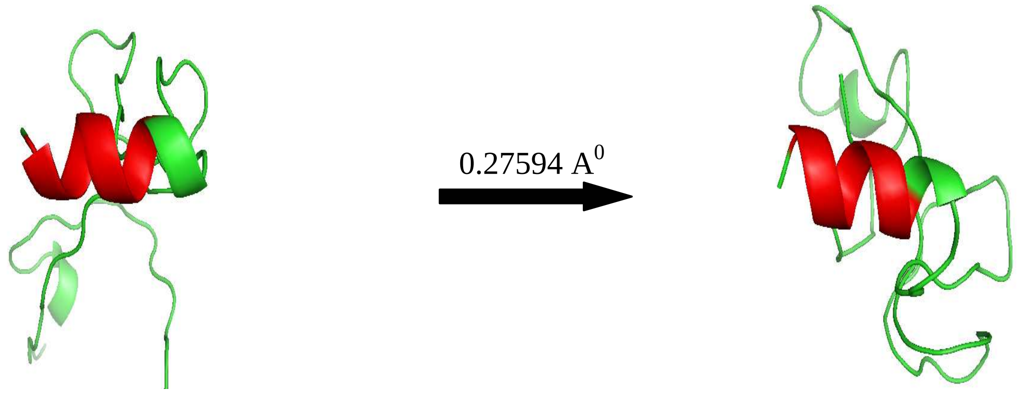

1HQ6A

Small segment 2-8 (ELDAKLN) of protein chain 1HQ6A is a conserved helical sequence. The protein chain belongs to Alpha & Beta Proteins (A+B) class of SCOP. The structure shows very little deviation from its helical conformation after MD simulation.

x)

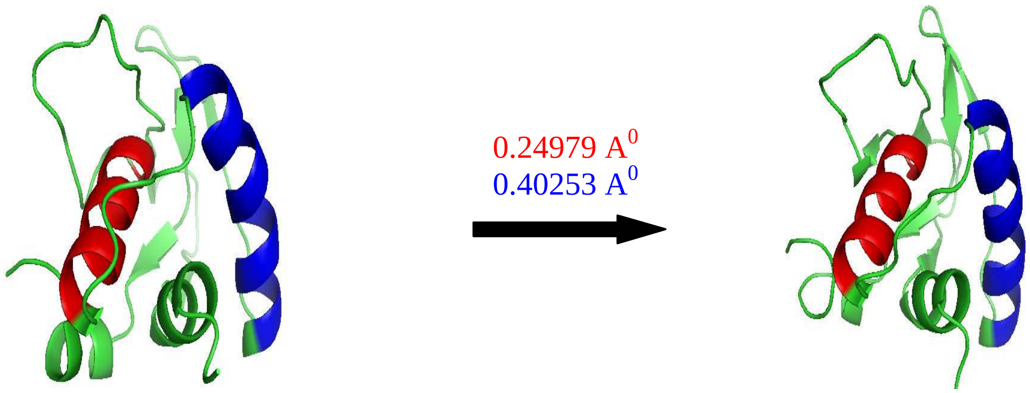

1KA8A

Protein chain 1KA8A has two conserved helical sequences: segment 37-47 (LYHAYLAYMEA) and segment 56-69 (LKMFGLGLPVMLKE). The protein chain belongs to All Alpha Proteins class of SCOP. Both the helical sequence is quite stable in nature which is evident from RMSD of initial and final structures of MD simulation of these segments.

xi)

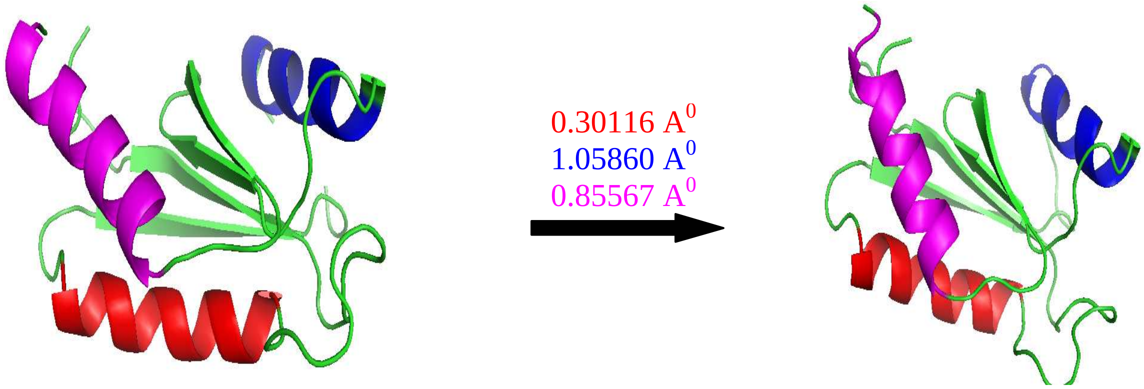

1XG8A

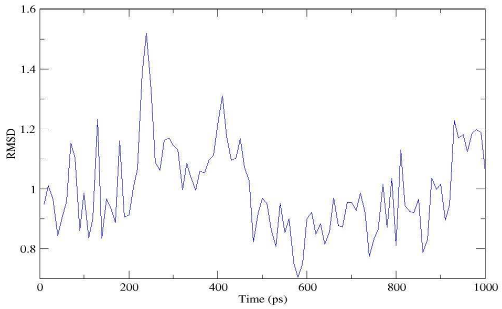

Three conserved helical sequences are present in protein chain 1X8GA: segment 19-33 (SKDIYDWLQPLLKRK), segment 55-65 (DHDLQFIERIE) and segment 88-101 (TKQITRFIDQKLVN). The protein chain belongs to Alpha & Beta Proteins (A/B) class of SCOP. Only the third segment shows a slight high RMSD between initial and final structure after simulation.

xii)

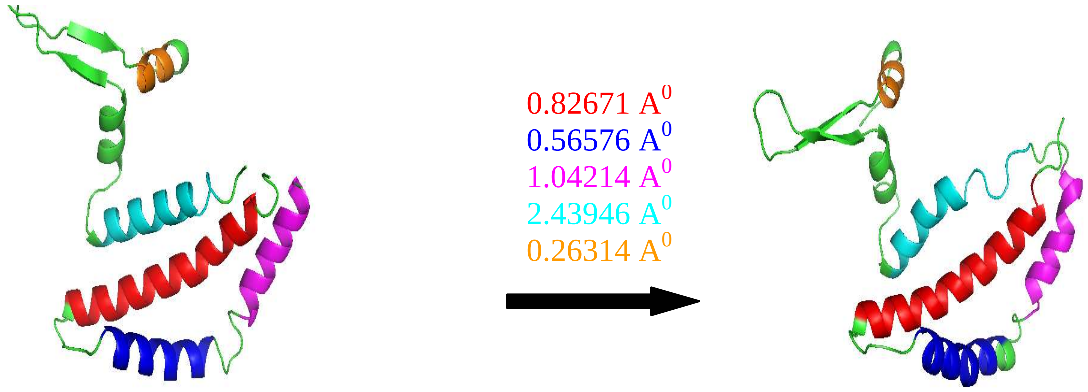

1KNZB

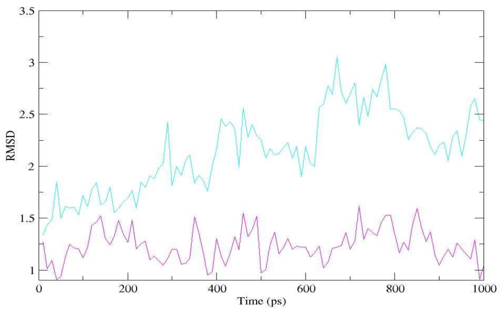

There are five conserved helical sequences in protein chain 1KNZB: segment 6-32 (STQQMAVSIINSSFEAAVVAATSALEN), segment 39-54 (YQDIYSRVKNKFDFVM), segment 61-74 (NNDIGKAITIDQAL), 93-110 (RPAKLDEDVNKLRMMLSS) and segment 141-147 (KLMRDKL). Third and fourth helix show some distortion which is also evident from RMSD of initial and final structures of these helical sequences after MD simulation.

xiii)

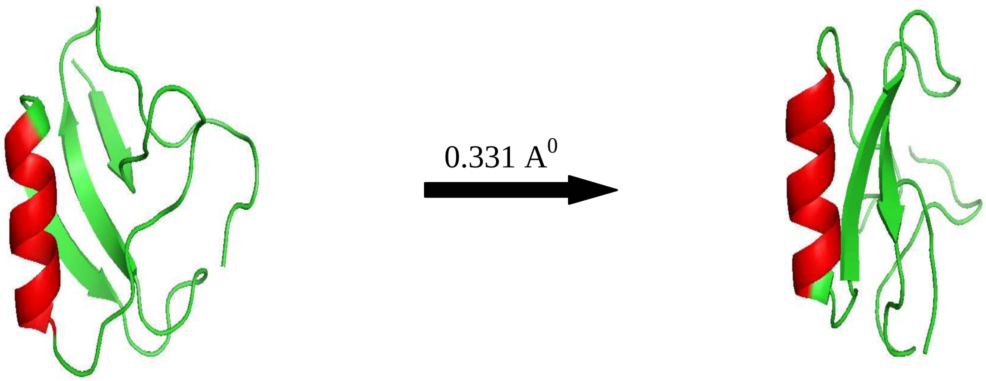

1AHOA

Segment 19-28 (NAYCNEECTK) of 1AHOA is a conserved helix. The protein chain belongs to Small proteins class of SCOP. After 1 nano second simulation the helical structure remains intact.
